# Genome-Wide Assessments Reveal Extremely High Levels of Polymorphism of Two Active Families of Mouse Endogenous Retroviral Elements

**DOI:** 10.1371/journal.pgen.1000007

**Published:** 2008-02-29

**Authors:** Ying Zhang, Irina A. Maksakova, Liane Gagnier, Louie N. van de Lagemaat, Dixie L. Mager

**Affiliations:** 1 Terry Fox Laboratory, B.C. Cancer Research Centre, Vancouver, British Columbia, Canada; 2 Department of Medical Genetics, University of British Columbia, Vancouver, British Columbia, Canada; The Jackson Laboratory, United States of America

## Abstract

Endogenous retroviral elements (ERVs) in mice are significant genomic mutagens, causing ∼10% of all reported spontaneous germ line mutations in laboratory strains. The majority of these mutations are due to insertions of two high copy ERV families, the IAP and ETn/MusD elements. This significant level of ongoing retrotranspositional activity suggests that inbred mice are highly variable in content of these two ERV groups. However, no comprehensive genome-wide studies have been performed to assess their level of polymorphism. Here we compared three test strains, for which sufficient genomic sequence is available, to each other and to the reference C57BL/6J genome and detected very high levels of insertional polymorphism for both ERV families, with an estimated false discovery rate of only 0.4%. Specifically, we found that at least 60% of IAP and 25% of ETn/MusD elements detected in any strain are absent in one or more of the other three strains. The polymorphic nature of a set of 40 ETn/MusD elements found within gene introns was confirmed using genomic PCR on DNA from a panel of mouse strains. For some cases, we detected gene-splicing abnormalities involving the ERV and obtained additional evidence for decreased gene expression in strains carrying the insertion. In total, we identified nearly 700 polymorphic IAP or ETn/MusD ERVs or solitary LTRs that reside in gene introns, providing potential candidates that may contribute to gene expression differences among strains. These extreme levels of polymorphism suggest that ERV insertions play a significant role in genetic drift of mouse lines.

## Author Summary

The laboratory mouse is the most widely used mammal for biological research. Hundreds of inbred mouse strains have been developed that vary in characteristics such as susceptibility to cancer or other diseases. There is much interest in uncovering differences between strains that result in different traits and, to aid this effort, millions of single nucleotide differences or polymorphisms between strains have been cataloged. To date, there has been less emphasis placed on other sources of genetic variation. In this study, we have conducted a genome-wide analysis to examine the level of polymorphism of mouse endogenous retroviral sequences (ERVs). ERVs are derived from infectious retroviruses that now exist in the genome and are inherited as part of chromosomes. Unlike in humans, genomic insertions of ERVs cause many new mutations in mice but their extent of variation between strains has been difficult to study because of their high copy numbers. By comparing genomic sequences of four common mouse strains, we found very high levels of polymorphism for two large active families of ERVs. Moreover, we documented nearly 700 polymorphic ERVs located within gene introns and found evidence that some of these affect gene transcript levels. This study demonstrates that ERV polymorphisms are a major source of genetic variability among mouse strains and likely contribute to strain-specific traits.

## Introduction

The laboratory mouse is the model of choice for mammalian biological research and a plethora of mouse genomic resources and databases now exist [1]. Notably, fueled by availability of genomic sequence for the common strain C57BL/6J (B6)[2], several groups have documented genetic variation among strains using single nucleotide polymorphisms (SNPs) [3]–[5]. Surveys of mouse polymorphism due to segmental duplications or copy number variations have also recently been published [6],[7]. Such resources are invaluable in trait mapping, in tracing strain origins and in genotype/phenotype studies. However, to date, genome-wide studies to document other types of genetic variation have been lacking. For example, long terminal repeat (LTR) retrotransposons/endogenous retroviral elements (ERVs) are known to be highly active in inbred mice, causing ∼10% of spontaneous mutations [8], but relatively little is known about the level of polymorphism of such sequences. Southern blotting and extensive genetic mapping has clearly demonstrated that ERVs related to murine leukemia virus (MLV) are highly polymorphic [9]–[11], but such techniques are feasible only for low copy number ERVs which constitute a very small fraction of ERVs and LTR retrotransposons in the mouse genome. Due to the array-based technology employed, the largest mouse polymorphism study performed by Perlegen focused only on SNPs and was not designed to detect insertional ERV polymorphisms [5].

Compared with a single nucleotide difference, genetic variation due to insertion of an ERV obviously has a much greater probability of affecting the host. Not only is the absolute change in the DNA much larger, but the inserted ERV sequences also carry powerful transcriptional regulatory elements that can influence host genes. The phenotypes of most mouse germ-line mutations caused by ERV insertions result not from simple physical disruption of coding regions, although this does occur, but rather from transcriptional abnormalities mediated by ERVs located in introns or near the affected genes [8]. It is also well appreciated that retroviruses can activate oncogenes or growth control genes leading to malignancy [9],[12],[13], and indeed, are used as tags to identify genes involved in cancer [14],[15]. Determining the extent of mouse ERV polymorphism is therefore critical in understanding how ERVs contribute to diversity and disease susceptibility among inbred strains.

The retroviral-like Intracisternal A Particle (IAP) and the MusD/Early Transposon (ETn) families are two high copy number ERVs responsible for most of the insertional germ-line mutations described in mice. IAP elements have been extensively studied since the early 1980s [16] and cause both germ line mutations as well as oncogene or growth factor gene activation in somatic cells [8],[9],[12],[17],[18]. ETn elements were also originally reported in the early 1980s as a non-coding transposon-like sequence expressed in early embryogenesis [19]–[21] and capable of causing new mutations. It is now known that ETns represent a non-coding subclass of the retroviral-like MusD elements [22],[23], which provide the proteins in *trans* necessary for ETns to retrotranspose [24]. Thus, throughout this study, this group is referred to as ETn/MusD elements.

According to a list compiled at the end of 2005 [8], six strain polymorphisms and 26 mutations due to insertions of IAP elements have been documented. Four polymorphisms and 19 mutations due to insertions of members of the ETn/MusD family have also been reported. Genomic hybridization and PCR methods have demonstrated that the IAP [25]–[27] and ETn/MusD [28] families are polymorphic among strains, but the extent of this variation and the potential consequences on phenotype are unknown. The goal of this work was to conduct a genome-wide assessment of the level of insertional polymorphism of the IAP and ETn/MusD families. A second goal was to identify polymorphic ERVs with the highest probability of affecting host genes. By comparing only the few strains for which sufficient genomic sequence is available, we found high levels of insertional polymorphism for both the IAP and ETn/MusD families. Moreover, we detected 695 polymorphic members of these families located within genes, and found evidence that some of these affect gene transcription. Such polymorphisms represent a substantial source of genetic variability among inbred strains and may play a major role in strain-specific traits.

## Results/Discussion

### Prevalence of ETn/MusDs and IAPs in Different Strains

As the first step to assess the ERV polymorphisms in mice, we conducted a survey of the overall copy numbers of IAP and ETn/MusD elements in the well-sequenced, assembled B6 genome using BLAST (see Materials and Methods for details). For the IAP family, we detected 2595 full-length or partly deleted elements plus 2477 solitary LTRs, for a total of 5072. ETn/MusD elements are less numerous than IAPs, with 1873 sequences in the B6 genome, 1457 of which are solitary LTRs. In accord with previous studies [29], our results indicated that solitary LTRs, the result of recombination between the 5′ and 3′ LTRs of proviral forms, are typically more common than full length ERVs.

For mouse strains other than B6, sufficient whole genome shotgun sequence traces (see Figure S1) are available for only three of them: A/J, DBA/2J, and 129X1/SvJ (referred to hereafter as the three test strains). To identify all traces containing IAP or ETn/MusD sequences, we used specifically designed ERV probes (see Figure S2 and Table S1) to screen the trace archives of the three strains with local sequence alignment. Sequences flanking the ERV segment in each trace were then used to map the region to a unique position in the assembled B6 genome and to combine redundant traces (see Materials and Methods). This screening method identified 1659, 1509 and 1379 ETn/MusD elements that could be assigned a unique location in A/J, DBA/2J and 129X1/SvJ, respectively. Similarly, for the IAP elements, we identified 4696 elements in A/J, 4320 in DBA/2J, and 3878 in 129X1/SvJ. As discussed above, our genomic survey detected 1873 MusD/ETn elements and 5072 IAPs in assembled B6 genome. The lower ERV numbers detected in the three test strains compared with B6 is likely mainly due to incomplete sequence coverage of the traces available for each strain. Another factor that contributes to the loss of detectable ERV insertions is inability to map the trace to a unique location, usually because the flanking non-ERV portion is too short, composed of other types of repeats, or is located within duplicated genomic regions. To determine the approximate fraction of elements from each of the three test strains that are not detectable due to incomplete sequence coverage or other reasons, we determined how many elements in the assembled B6 genome could be found with our method using randomly sampled sets of WGS traces from the B6 trace archive database. Using numbers of B6 traces equivalent to that available for A/J (11,646,236), DBA/2J (7,998,826) and 129X1/SvJ (5,998,950), we detected 83.8%, 77.9% and 68.6% of the 1865 ETn/MusD insertions present in the assembled B6 genome (Figure S3). Thus, it seems reasonable that approximately 16.2%, 22.1% and 33.4% of the ERVs present in the three test strains are not found due to incomplete coverage or mapping difficulties. Moreover, this B6 trace sampling experiment also allowed us to conservatively estimate the false discovery rate of this procedure to be ∼0.4% (see Materials and Methods).

### Identification and Frequency of Polymorphic ERVs

**Figure 1:**
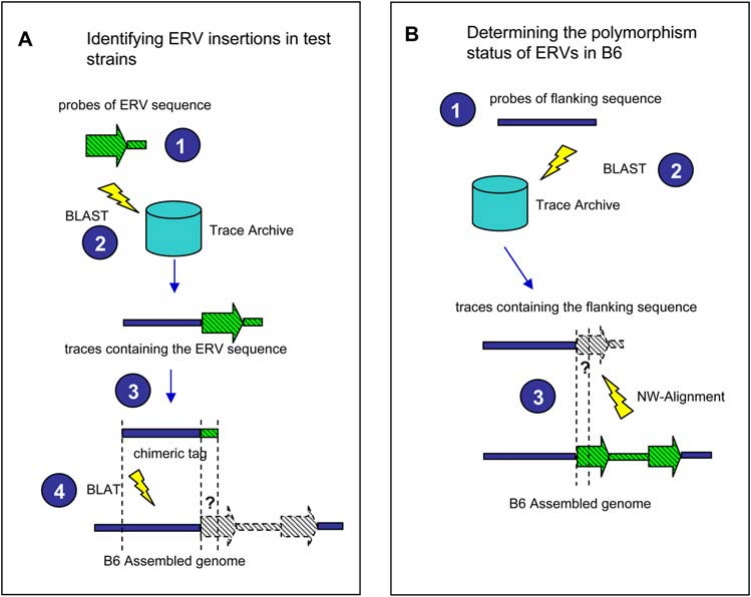
Screening strategy for detection of polymorphic ERV insertions. A) Identification of ERV insertions in test strains. In the first step, ERV probes of different lengths were designed based on known ERV sequences (see Materials and Methods and Table S1 for more details). Next, the ERV probes were aligned to trace sequences of the test strain with WU-BLAST, and all traces containing the target ERV sequences were retrieved (step 2). From each ERV-containing trace, a chimeric tag was constructed by taking the flanking genomic sequence appended with a small tail (≤50 bp) of the target ERV sequence (step 3). In the final step, all chimeric tags were mapped to the assembled B6 genome with BLAT, and the existence of corresponding ERVs in B6 was determined by checking whether the small ERV-tail was included in the alignment (step 4). B) Determining the polymorphism status of ERVs present in B6. In the first step, probes were built based on the sequences flanking all ERV insertions in the B6 genome. In the next step, these probes were used to select all traces containing such flanking sequences in test strains. In the third step, a 35-bp-region adjacent to the mapped flanking sequence in each trace obtained from previous step was compared to the corresponding ERV sequence in the B6 genome, and the existence of such ERV element in the test strain was assessed according to the sequence identity. In both panels, solid blue bars represent genomic sequences flanking the ERV insertions in mice. Green hatched bars or arrows with solid borders are ERV internal or LTR sequences, respectively. Gray shaded bars or arrows with broken borders are suspected ERV sequences, of which the existence is determined by the alignment score of regions annotated with “?”s.

As outlined in Figure 1 and described fully in Materials and Methods, we designed a four-phase screening process to identify polymorphic ERVs. In the first phase, probes derived from known ERV sequences were used to screen the B6 assembled genome and a collection of ETn/MusD or IAP elements in B6 was obtained. In the second phase, illustrated in Figure 1A, we determined if the ERVs identified in the three test strains were also present in B6 by checking for existence of such ERV sequences at corresponding loci in the assembled B6 genome. In the third phase, represented in Figure 1B, we included the dataset of all ERVs present in B6 and determined the presence of these ERVs in the three test strains. To achieve this, we retrieved the 5′ and 3′ flanking sequences from elements present in the assembled B6 genome, obtained those flanking segments that could be uniquely mapped to the genome and then identified sequence traces from the test strains that contain these flanking segments. The traces were then checked for presence of the ERV. In the final phase, a similar strategy was applied to the polymorphic ERV insertions found in each test strain (but not in B6), and the existence of corresponding ERVs in the other two test strains was assessed. The combination of these strategies allowed us to compile lists of ERV genomic locations and the polymorphism status of each ERV in the four strains. Due to inability to uniquely map many ERV flanking regions to the short, unassembled sequence traces, the status of many elements present in the assembled B6 genome could not be computationally determined in the test strains (see below). In addition, as discussed above, incomplete sequence coverage of the test strains results in an “unknown” status for a proportion of ERVs in each test strain.

**Figure 2:**
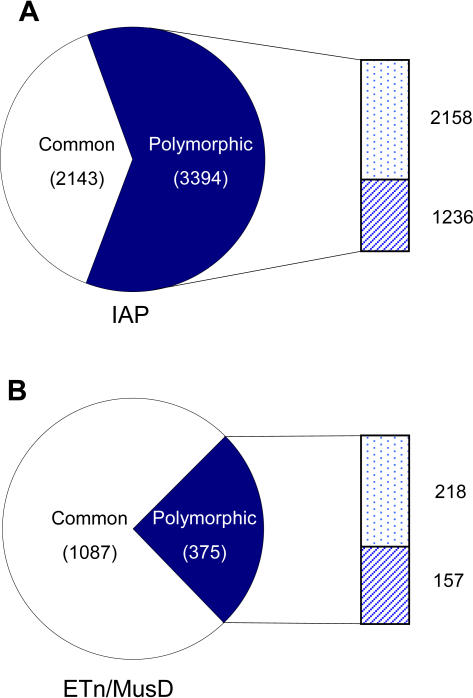
Fractions of polymorphic ERVs based on the four strains. Pie charts indicate the status of all detectable IAP elements (Part A) and ETn/MusD elements (Part B). White sections indicate the fraction of elements that could be scored as present in all four strains (annotated as ‘common’). Dark blue sections represent the fraction of elements scored as absent in at least one strain (annotated as ‘polymorphic’). Side bars illustrate the data composition of polymorphic ERVs, with dotted/striped sections indicating polymorphic elements for which status could be/not be confirmed in all four strains, respectively.

In spite of these limitations, we identified a large number of polymorphic ERVs (Figure 2). Of all IAP elements detected in at least one strain, 2143 were present in all four strains while 3394 elements were scored as polymorphic (absent in at least one of the four strains), giving an overall polymorphic fraction of 61.3%. For ETn/MusD elements, 1087 were mapped as present in all four strains and 375 could be scored as absent in one or more strains, a polymorphic fraction of 25.6% of all the elements having a determinable status. Another 1767 IAP and 660 ETn/MusD elements present in the assembled B6 genome could not be mapped to the test strain traces due to incomplete trace coverage or repetitive flanking regions, so their polymorphic status could not be computationally determined. These high levels of insertional polymorphism were obtained by considering just four strains and despite the fact that the status of many elements could not be ascertained in some strains. Thus, the numbers of polymorphic ERVs among inbred mice must be significantly higher.

### Genic Distribution Patterns of the Youngest ERVs are Distinct from Older Elements

Previous studies on human ERVs have shown that they are less prevalent in gene introns than expected by chance, likely due to selection against LTR elements found in genes [30]–[32]. Although they can affect genes at significant distances [9],[13],[17], retroviral elements or LTRs in introns are more likely to impact expression by introducing powerful transcriptional regulatory elements and splice sites [32]. Moreover, genomic analyses in several species have shown that ERVs/LTRs in introns are more likely to be oriented antisense to the enclosing gene [30]–[34]. Since retroviruses show no orientation bias upon insertion into genes (i.e. 50% in sense direction) [34],[35], this antisense bias is likely the result of stronger negative selection against sense-oriented elements. Indeed, of the 19 cases of ETn elements known to disrupt gene expression in various new mutations, 16 are oriented in the same direction as the gene [8],[32], indicating that sense-oriented elements are much more likely to perturb gene expression, causing a detectable phenotype, and being subject to negative selection. Although the original integration site preferences for ERVs are generally unknown, two studies have mapped small sets of fresh (unselected) insertions of IAP and ETn/MusD elements in retrotransposition assay systems and the data are consistent with a fairly random pattern of integration and no strand bias upon insertion into transcriptional units [24],[36].

**Figure 3:**
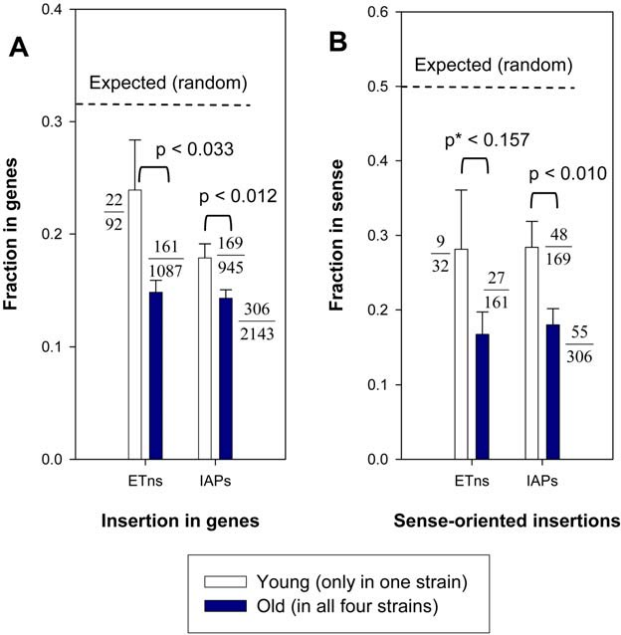
Distributions of young versus older ERV elements with respect to genes. A) Fraction of elements located within genes. B) Fraction of genic elements oriented in the same transcriptional direction as the gene. Dark blue bars represent elements found in all four strains. White bars represent ERVs present in only one of the four strains. Dashed lines indicate the expected fractions assuming a random genomic integration pattern. Error bars show standard errors, and P-values based on two sample z-test comparing young and old groups are shown. All comparisons between the “young” and “old” subsets are statistically significantly different except for the orientation bias of ETn/MusD elements (marked with “*” in Figure 3B), due to the low numbers of elements in this category. Actual numbers of elements in each category are shown as numerators in fractions, with denominators being the total numbers of elements in the different groups.

Given that the genomic distributions of ERVs fixed in a species are strongly shaped by selection, we predict that recently inserted ERVs will display genic distributions different from their older cousins. To test this prediction, we compared the distributional properties of a subset enriched for the youngest ERVs with that of ERVs common to all four strains. To obtain the youngest elements, we chose those present in only one strain and which could be computationally scored as absent in the other three strains. Many of these likely still represent older polymorphic elements due to the fact that lab strains are genetic mixtures of subspecies of *Mus* [3]–[5],[37]. However, this group will contain all the truly young elements that inserted after strain divergence. As shown in Figure 3, these datasets enriched for the “youngest” elements are more likely to be found in genes (Figure 3A) and in the sense orientation within genes (Figure 3B), compared with elements shared between all four strains. The higher prevalence in genes and reduced intronic orientation bias displayed by ERV subsets enriched for the youngest elements suggests that some are deleterious but have inserted very recently and have not been eliminated by selection.

### Confirmation of Polymorphic ERVs in Gene Introns

Our bioinformatics screens identified 623 polymorphic IAP elements and 72 polymorphic ETn/MusD elements located within genes in one or more of the four strains. Complete lists of these elements and their locations with respect to the B6 genome are given in Tables S2 and S3. These tables list in which of the four strains each element was computationally detected by our screens. As discussed above, the question marks in the Tables are mainly due to mapping difficulties or incomplete sequence coverage of the trace databases. A subset of these elements was analyzed using genomic PCR on DNA from a panel of mouse strains (including B6 and the three test strains) with primers flanking the insertion site to verify the insertion status. For this analysis, we chose all 28 cases of ETn/MusD elements found in A/J gene introns but absent in B6, and 12 cases of ETn/MusD elements present in B6 gene introns but scored as absent in A/J (Table 1). For the 28 cases of elements computationally detected in A/J (cases 1–28 in Table 1), the ETn insertion in the dysferlin (*Dysf* ) gene (case #9) is the only previously reported case and occurred 20–30 years ago in the A/J breeding stocks [38]. For the set of 12 elements present in B6 (cases 29–40 in Table 1), the ETn element in the *Wiz* gene (case #40) has also previously been reported as polymorphic [28].

**Table 1:** Genomic PCR verification of ETn/MusD intronic insertions in different mouse strains^a^.

case#	gene	location	intron size	insert_site b	orient. c	B6	B6(p)	A/J	A/J(p)	DBA	DBA(p)	129X1	129X1(p)	A/WySn	SWR/J	C3H	Balb	Size^f^
1	*2310035C23Rik*	intron 13	1924	1:107544558	A	**-** d	N^e^	**+** d	Y^e^	**-**	N	**-**	N	**+**	**+**	**+**	**+**	350
2	*Dnajc10*	intron 3	1920	2:80121109	S	**-**	N	**+**	Y	**-**	N	**-**	N	**+**	**-**	**-**	**-**	5400
3	*Atp9a*	intron 14	5914	2:168357724	A	**-**	N	**+**	Y	**-**	N	**-**	N	**-**	**-**	**-**	**-**	5700
4	*Gem*	intron 2	4896	4:11637425	A	**-**	N	**+**	Y	**-**	N	**-**	?	**+**	**-**	**-**	**-**	5500
5	*B230396O12Rik*	intron 6	2946	4:153708611	A	**-**	N	**+**	Y	**-**	N	**-**	?	**+**	**-**	**-**	**-**	5500
6	*Art3*	intron 2	8817	5:93471996	A	**-**	N	**+**	Y	**-**	N	**-**	?	F	**-**	**-**	**-**	5500
7	*Foxk1*	intron 1	33098	5:142663608	A	**-**	N	**+**	Y	**-**	N	**-**	N	**+**	**-**	**+**	**+**	5500
8	*Stk31*	intron 3	6516	6:49330234	A	**-**	N	**+**	Y	**-**	?	**-**	N	**+**	**-**	**-**	**-**	5500
9	*Dysf*	intron 4	4795	6:84024675	S	**-**	N	**+**	Y	**-**	N	**-**	?	**-**	**-**	**-**	**-**	6000
10	*Zfp82*	intron 5	4708	7:29768570	A	**-**	N	**+**	Y	**+**	?	**+**	?	**+**	**-**	**-**	**+**	1700
11	*Pde8a*	intron 12	1773	7:81189399	A	**-**	N	**+**	Y	**-**	N	**-**	N	**+**	**+**	**-**	**+**	400
12	*Pgbd5*	intron 1	49175	8:127312767	A	**-**	N	**+**	Y	**-**	N	**+**	?	**+**	**-**	**-**	**+**	400
13	*Opcml*	intron 2	270720	9:28170744	S	**-**	N	**+**	Y	**-**	N	**-**	N	**-**	**-**	**-**	**-**	5400
14	*Alg9*	intron 14	20017	9:50582311	A	**-**	N	**+**	Y	**+**	Y	**-**	N	**+**	**-**	**+**	**+**	unk.
15	*Zfp291*	intron 8	8166	9:55688688	A	**-**	N	**+**	Y	**+**	Y	**+**	Y	**+**	**-**	**+**	**-**	350
16	*Cdh23*	intron 32	2103	10:59779699	A	**-**	N	**+**	Y	**-**	N	**-**	N	**-**	**-**	**-**	**-**	5600
17	*Odz2*	intron 8	35382	11:36015943	A	**-**	N	**+**	Y	**-**	N	**-**	N	**+**	**-**	**-**	**+**	7500
18	*Prkca*	intron 3	134255	11:107934734	S	**-**	N	**+**	Y	**-**	N	**-**	?	**+**	**-**	**-**	**-**	5500
19	*Akap6*	intron 7	71966	12:53930690	A	**-**	N	**+**	Y	**-**	N	**-**	N	**+**	**-**	**+**	**+**	unk.
20	*Mark3*	intron 14	7429	12:112088888	A	**-**	N	**+**	Y	**-**	N	**-**	?	**+**	**-**	**-**	**-**	7800
21	*LOC432723*	intron 1	61826	13:4788783	A	**-**	N	**+**	Y	**-**	N	**+**	Y	**+**	**+**	**-**	**+**	350
22	*Cacna2d3*	intron 11	109422	14:28067384	A	**-**	N	**+**	Y	**-**	?	**+**	Y	**+**	**+**	**+**	**+**	400
23	*A2bp1*	intron 3	321339	16:6641077	S	**-**	N	**+**	Y	**+**	Y	**+**	Y	**+**	**+**	**+**	**+**	5500
24	*Sytl3*	intron 4	12675	17:6573500	S	**-**	N	**-**	Y	**-**	N	**-**	?	**-**	**-**	**-**	**-**	N/A
25	*Dlgap1*	intron 4	55340	17:70603001	A	**-**	N	**+**	Y	**+**	Y	**+**	Y	**+**	**+**	**+**	**+**	350
26	*Dym*	intron 16	43220	18:75389795	A	**-**	N	**+**	Y	**-**	N	**-**	N	**-**	**-**	**-**	**-**	5700
27	*Mtm1*	intron 8	4446	X:67552081	S	**-**	N	**+**	Y	**-**	N	**-**	?	**+**	**-**	**+**	**+**	5500
28	*Col4a6*	intron 2	146075	X:136618162	S	**-**	N	**+**	Y	**-**	N	**+**	Y	**+**	**-**	**-**	**+**	5500
29	*Sh3bp4*	intron 1	36659	1:90927936	A	**+**	Y	**-**	N	**-**	N	**-**	N	**-**	**-**	**+**	**+**	5550
30	*Tor3a*	intron 4	10001	1:158497287	A	**+**	Y	**-**	N	**-**	N	**+**	?	**-**	**+**	**-**	**-**	330
31	*Cd84*	intron 2	20560	1:173693834	A	**+**	Y	**-**	N	**-**	N	**-**	?	**-**	**-**	**-**	**-**	1574
32	*Ttbk2*	intron 4	16402	2:120487764	A	**+**	Y	**-**	N	**-**	N	**-**	N	**-**	**-**	**-**	**-**	5478
33	*Unc13b*	intron 7	46281	4:43167395	A	**+**	Y	**-**	N	**-**	N	**+**	Y	**-**	**-**	**-**	**-**	322
34	*Cadm4*	intron 1	16992	7:24206094	A	**+**	Y	**+**	N	**+**	Y	**+**	Y	**+**	**+**	**+**	**+**	338
35	*Dpep1*	intron 1	7590	8:126077748	A	**+**	Y	**-**	N	**+**	Y	**-**	N	**-**	**+**	**+**	**-**	322
36	*Vnn3*	intron 3	7946	10:23546116	A	**+**	Y	**-**	N	**+**	Y	**+**	Y	**-**	**-**	**-**	**-**	5696
37	*Slfn8*	intron 4	8583	11:82825929	A	**+**	Y	**-**	N	**-**	Y	**-**	N	**-**	F	**-**	**+**	334
38	*Klhl1*	intron 8	15107	14:95020963	A	**+**	Y	**-**	N	**-**	?	**+**	Y	**-**	**-**	**-**	**-**	320
39	*Mapk14*	intron 6	2511	17:28455067	A	+	Y	-	N	F	Y	+	Y	-	-	-	-	320
40	*Wiz*	intron 2	19667	17:32100856	A	+	Y	-	N	-	N	-	N	-	-	-	-	7121

a Full names of mouse strains are given in Materials and Methods. A (p) after strain names indicates computational predictions.

b Corresponding position in B6 genome (version mm8).

c A: insertion is antisense to gene; S: insertion is sense to the gene.

d + indicates presence of insertion; - indicates absence of insertion; “F” indicates PCR failure.

e Y: computational prediction of having insertion; N: computational prediction of no insertion; ?: presence of insertion could not be determined computationally.

f Size of ERV insertion (approximate for cases 1–28).

**Figure 4:**
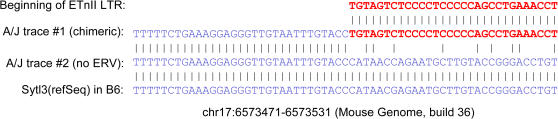
An apparent heterozygous ETn insertion in the *Sytl3* gene in an A/J mouse. The top line is the first 30bp of an ETnII LTR. The second line is from the A/J trace *gnl|ti|1104656312*, which consists of a non-ERV part and an ETn LTR part. The third line is a different trace sequence from A/J (*gnl|ti|1344398576*). The bottom line is from the RefSeq gene *Sytl3* in the assembled B6 genome (build 36) with genomic coordinates shown. ETn sequences are bold red, and non-ETn sequences are blue.

In total, these 40 selected cases and four strains generated an experimental space of 160 predictions. As shown in Table 1, columns with a strain name followed by a “(p)” indicate that data in these columns are computational predictions of the existence of the ERV insertions in the corresponding strain. After excluding 16 undeterminable instances (denoted as “?”s in these columns in Table 1), we computationally determined the presence of these ERV insertions in all four strains with a total number of 144 predictions. For 140 of these, our computational predictions precisely matched the experimental confirmation of ERV insertion status using genomic PCR, demonstrating a high accuracy of our bioinformatics screens. In one instance, (case #39 in DBA), the PCR failed so we could not test our prediction. Therefore, only three cases showed anomalous PCR results that did not match our bioinformatics predictions. One of these cases was #24 in Table 1, where we predicted an ETn/MusD insertion in an intron of the *Sytl3* gene in A/J mice. Using PCR, we found no evidence for this insertion in the A/J DNA sample used. We then reexamined the A/J sequence dataset and found orthologous sequence traces both with and without this particular ERV element (Figure 4). The most likely explanation for this finding is that this ERV represents a very recent insertion present in a heterozygous state in the A/J genomic DNA used to generate the trace sequence data. Since the rate of ETn/MusD retrotransposition in A/J is relatively high compared with other strains [8], it is not surprising that individual A/J mice will have occasional “private” insertions. The second anomalous case was #34 of an ETn/MusD LTR found in the B6 genome within the *Cadm4* gene, and confirmed as present in all tested strains by PCR (Table 1). Our computational screens correctly scored this LTR as present in DBA/2J and 129X1/SvJ but scored it as absent in A/J. Upon further examination of the sequence data, we found that one of the two available A/J sequence traces mapping to this location is an artifact since it contains a segment of unknown origin. The other trace is also unusual as segments of it map to two locations several kb apart. Thus, this case can be explained by artifactual sequence traces, demonstrating that the trace archives and, therefore, our dataset are not without errors. The last inconsistent case was #37, an insertion located within the *Slfn8* gene and predicted as present in both B6 and DBA. In this case, the PCR verification in DBA showed that the element is not present. Since both the computational and experimental results were clear yet contradictory, we do not have a definitive explanation for this case, although it is possible the trace is not of DBA origin. In any event, this case was regarded as a false positive. In several instances, the PCR data also allowed us to assign a definite insertion status to elements in test strains that could not be predicted *in silico* due to incomplete sequence coverage of the traces (see Table 1).

As expected, some of these insertions are not specific to a single strain. This finding indicates that many of the polymorphic ERV insertions arose prior to divergence of common inbred strains or represent even older polymorphisms due to different origins of chromosomal segments in the genomes of today's lab mice. For the 28 cases present in A/J but absent from B6, the short A/J sequence traces do not contain the entire ERV, but length of the inserted element could be estimated from the size of the genomic PCR product for 25 of these cases (see last column in Table 1). In 15 cases, the size matches that expected for an ETn element of 5.5–6 kb, whereas two appear to be full length MusD elements of 7.5–7.8 kb and one is likely a partly deleted element (case #10). Seven are solitary LTRs (320–400 bp), so the nature of the original insertion cannot be determined since the LTRs of ETnII elements and MusDs are extremely similar [22],[39]. For the 12 elements present in the assembled B6 genome, seven are solitary LTRs, one is a partial element and four are ETn elements based on size and sequence. The element in the *Wiz* gene is a longer ETn variant [28]. The preponderance of polymorphic ETn elements over MusD was expected, given that most published mutagenic insertions of this family are of the ETnII subfamily [8],[28].

### Potential Gene Expression Effects Mediated by Polymorphic ERVs

Since ERVs/LTRs can affect gene transcription via a variety of mechanisms, some of the polymorphic ERVs detected here may contribute to gene expression differences between strains, possibly leading to phenotypic differences. However, the factors that determine whether transcription of a gene will be affected by a nearby or intragenic ERV insertion are not understood and are likely complex. Thus, it is not possible to estimate what fraction of the polymorphic insertions documented here may have functional consequences. Nonetheless, we can predict which cases may be more likely to affect gene expression. In the majority of documented cases where a new mutagenic ETn/MusD insertion causes significant transcriptional defects, the element has been located within an intron in the sense orientation and disrupted splicing patterns of the gene [8]. Thus, we predict that ETn elements within introns and oriented in the same direction as the enclosing gene have a relatively high probability of affecting mRNA processing. Moreover, compared with older insertions, the youngest, polymorphic subsets of these elements are potentially more likely to impact host gene expression, as selection may still be operating in these cases.

**Figure 5:**
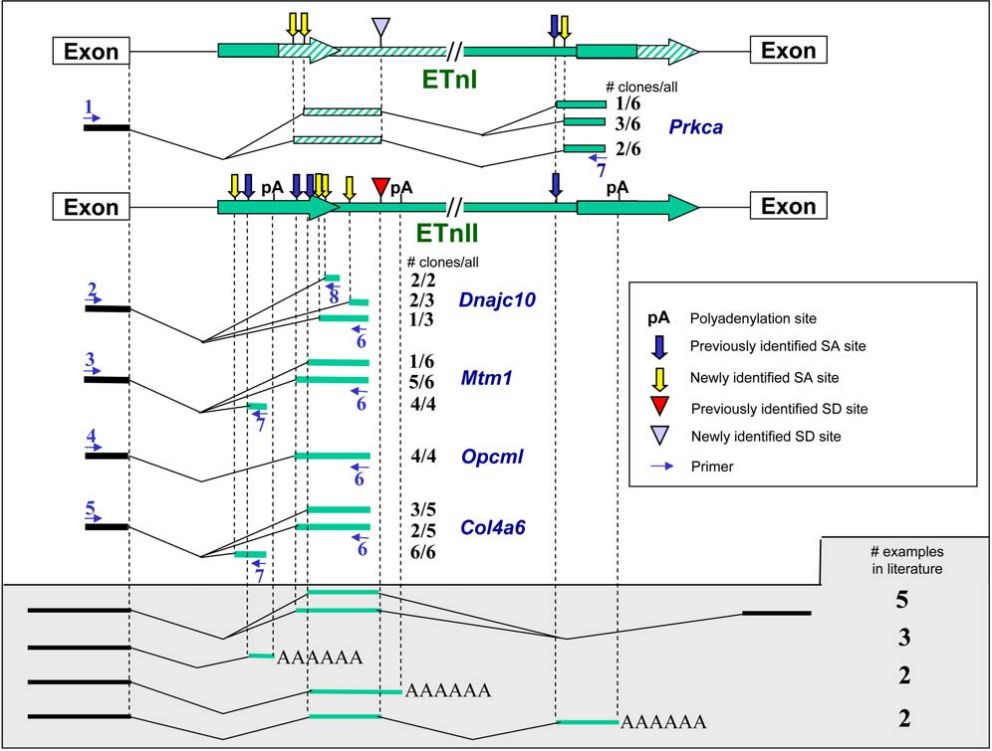
Detection of ETn-gene chimeric transcripts. Aberrant transcripts induced by a novel ETn insertion into a gene intron are shown. Gene direction, from left to right, is the same as ETn orientation. Genes *Dnajc10, Opcml, Mtm1* and *Col4a6* harbor ETnII insertions, while *Prkca* has an ETnI insertion. ETn sections that are different in an ETnI element compared to ETnII are shown as striped. Cryptic and natural splice acceptor sites are designated as blue (previously identified) or yellow (newly identified in this study) vertical arrows. Splice donor sites are represented as red (previously known) or light blue (newly identified) triangles. Natural and cryptic polyadenylation sites are marked as pA. Numbered thin arrows denote primers used to amplify chimeric transcripts. Sense primers in the upstream exon are as follows: 1, Prkca-up-ex-s; 2, Dnajc10-up-ex-s; 3, Mtm1-up-ex-s; 4, Opcml-up-ex-s; 5, Col4a6-up-ex-s. Antisense primers in the ETn are as follows: 6, IM_3as; 7, MusD2_7130as; 8, LTR_2as. Number of clones for each transcript variant compared to all clones sequenced for each primer pair is indicated. Chimeric ETn transcripts and number of their examples identified previously [8] are shown at the bottom. Sequences of all splice sites used in these cases are shown in Figure S4.

Based on the above reasoning, we chose a subset of cases to examine further using the following criteria: First, since the consequences of IAP insertions can involve LTR bidirectional promoter effects [8] which are more complicated and difficult to predict, we focused on ETn/MusD insertions. Second, we chose intronic ETn elements oriented in the same direction as the gene. Third, we chose elements verified as present in A/J and lacking in B6 using genomic PCR (see Table 1). Seven such cases exist, involving ETns in the *Dnajc10, Dysf, Opcml, Prkca*, *A2bp1, Mtm1,* and *Col4a6* genes. We performed RT-PCR on RNA from A/J mice using primers from the gene exon upstream of the ETn insertion, coupled with primers from within the ETn, chosen to detect the most frequently reported types of ETn-mediated transcriptional fusions from the literature [8]. Sources of RNAs were chosen based on known expression patterns of the gene. As shown in Figure 5, chimeric transcripts were detected for all five of the genes tested, namely *Dnajc10, Prkca*, *Mtm1, Opcm1* and *Col4a6.* The sense-oriented ETn element found in the *Dysf* gene in A/J has already been shown to cause similar splicing defects [38] and we did not examine *A2bp1.* In most cases, the splice sites used in the ETn element in the examples analyzed here were analogous to those characterized in known mutagenic cases. However, for *Prkca*, this analysis showed that the insertion is a member of the ETnI subfamily, as opposed to ETnII, and revealed usage of cryptic splice acceptor sites not previously documented. (see Figure S4 for sequences of splice sites). It should be noted that the subset of chimeric transcripts shown in Figure 5 is likely an underestimate, since a limited number of clones were sequenced and not all transcript variants would have been detected with the primers used. This RT-PCR analysis demonstrates that these ETn elements cause patterns of aberrant splicing similar to those documented in cases of known mutations due to new ETn integrations. However, further quantitative analyses are required to determine the significance of these splicing abnormalities in affecting overall levels of gene expression. Such in depth experimental investigations for each case are beyond the scope of the present study.

**Figure 6:**
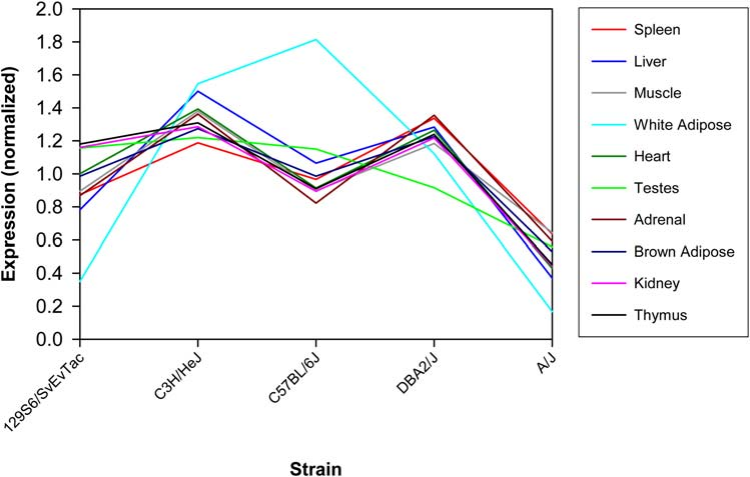
Normalized, tissue-averaged expression of *Dnajc10* across strains. Analysis of microarray data of Zapala et al [41] (NCBI GEO accession GSE3594) of transcript levels in 10 tissues profiled in A/J, B6, C3H/HeJ, DBA/2J and 129S6/SvEvTac mice. Replicates within a tissue/strain were averaged and a variation pattern of relative expression levels across the above five strains was computed for each tissue. These patterns were found to be similar across tissues (except white adipose tissue which was an outlier).

We also surveyed microarray data on gene expression differences in inbred strains available through the Gene Expression Omnibus [40] (http://www.ncbi.nlm.nih.gov/geo/). We examined all cases listed in Table 1 for correlations between presence of the insertion and differences in gene transcript levels compared with strains lacking the insertion (see Materials and Methods). Specifically, we analyzed the microarray data of Zapala et al. [41] (NCBI GEO accession GSE3594) that includes data on gene expression in 10 tissues profiled in A/J, B6, C3H/HeJ, DBA/2J and 129S6/SvEvTac mice. For the *Dnajc10* gene, tissue-wide reduction in expression was noted in A/J mice relative to the other four strains (p<10^−4^, Binomial distribution) (Figure 6). Microarray data available through the GeneNetwork web site (http://www.genenetwork.org/) also showed that transcript levels of this gene in A/J are much lower than in all other tested strains, based on whole brain, cerebellum, hippocampus and eye datasets (Figure S5). *Dnajc10* has a sense-oriented ETn element in the third intron in A/J and the related A/WySn mice, but no other tested strain (Table 1). This gene (also termed *ERdj5*) encodes an endoplasmic reticulum (ER) chaperone protein induced during ER stress and is likely involved in protein folding [42],[43].

**Figure 7:**
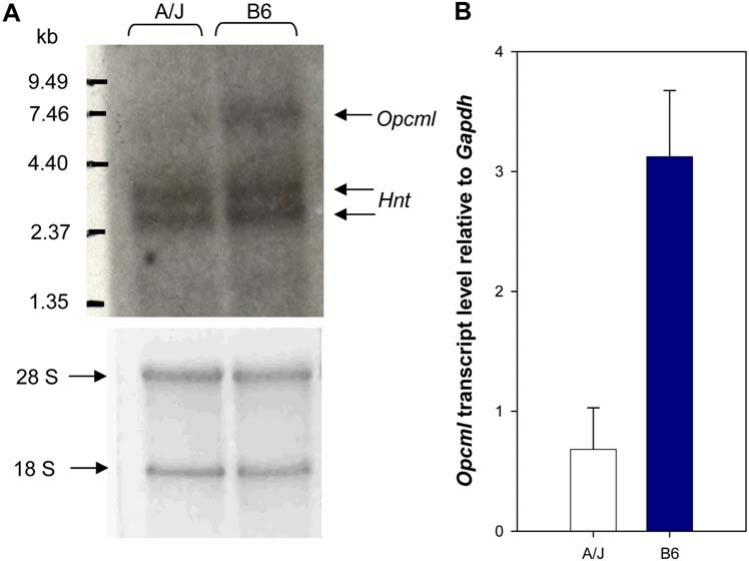
Transcript levels of *Opcml* in A/J versus B6. A) Northern blotting. Total RNA from the cerebellum of an A/J and B6 mouse was hybridized to a probe from the *Opcml* exon upstream of the ETn insertion in A/J. The lower part of the figure is the ethidium bromide-stained gel, showing even loading as indicated by the ribosomal RNA bands. The band corresponding to *Opcml* is marked with an arrow. The probe cross-hybridized with a related gene *Hnt*, as explained in the text. B) Semi-quantitative PCR on cDNA from A/J and B6 cerebral hemispheres. *Opcml* cDNA was amplified with primers from upstream and downstream of the ETn insertion site. *Opcml* and *Gapdh* fragments were amplified from cDNA dilutions; for each dilution, the intensity of the resulting band was quantified and graphed as transcript levels of *Opcml* relative to *Gapdh* (see Figure S7). The average and standard deviation for all experiments is shown.

Another gene for which significant differences in expression correlate with presence of an ETn element is *Opcml*. No data is available from the Zapala et al. study on this gene but datasets accessed through GeneNetwork show that transcript levels in A/J, the only tested strain carrying an ETn insertion (Table 1), are significantly lower than in any other strain in cerebellum, whole brain, hippocampus and eye, the only tissues where A/J microarray information is available for this gene (Figure S6). *Opcml* (Opioid binding protein/cell adhesion molecule-like), also termed *Obcam*, is a member of the IgLON gene family and encodes a synaptic neural cell adhesion molecule [44],[45]. Loss of expression and/or promoter hypermethylation of this gene has been reported in some human cancers, suggesting that it may play a tumor suppressive role [46],[47]. We performed Northern blot analysis on total RNA from A/J and B6 cerebellum using a probe derived from the exon upstream of the insertion site and results are shown in Figure 7A. The ∼6.5 kb band corresponding to *Opcml* full length mRNA is markedly decreased in A/J compared with B6. A similar reduction in *Opcml* RNA was also observed in A/J using an exon probe downstream of the insertion site (data not shown). The two bands at 3–3.5 kb are due to cross-hybridization to another gene, neurotrimin (*Hnt*), which is a closely linked member of the IgLON family and highly related to *Opcml* in the region used as a probe [48]. We also performed semi-quantitative RT-PCR on total RNA from A/J and B6 cerebral hemispheres using primers from *Opcml* exons just upstream and downstream of the ETn insertion site and found an approximately 4.6-fold reduction in the correctly spliced *Opcml* RNA in A/J relative to B6 (Figure 7B). These results confirm the microarray data (Figure S6) and show that presence of the ETn insertion correlates with a substantial decrease in full length, correctly spliced *Opcml* mRNA. While there could be other reasons for the reduced transcript levels, such patterns suggest that the ETn element in these two genes significantly affects expression by causing aberrant splicing (as shown in Figure 5) allowing only a minor fraction of normal transcripts to be produced.

For all other cases from Table 1, including the other genes with insertions that cause aberrant splicing detected by RT-PCR (Figure 5), available microarray data was either inconsistent or did not show a clear relationship between presence of the insertion and altered levels of transcripts. These findings suggest that, in most cases, the ETn insertion has no significant effect on expression. This result is not surprising since thousands of ERVs or LTRs have become fixed during evolution in human and mouse genes [32], indicating that they can reside within introns without a functional impact. However, as illustrated by the *Dysf* case, the microarray data should be treated with caution. It has been convincingly shown by Northern analysis that A/J mice with the ETn insertion lack full length *Dsyf* mRNA and protein in skeletal muscle [38]. However, the available microarray data for *Dysf* is limited to cerebellum and whole brain, neither of which shows abnormally low transcript levels in A/J (data not shown). There could be several reasons for this discrepancy but it illustrates that wet lab approaches are necessary to properly evaluate each case.

Besides causing gene splicing defects similar to ETns, it is well established that IAP LTRs can also promote ectopic gene transcription in cases of somatic oncogene activations and germ line mutations [8],[9],[12],[17],[18]. Moreover, a few mutations caused by IAP-driven aberrant gene expression have been shown to act as metastable epialleles, exhibiting variable expressivity among genetic identical mice linked to the variable epigenetic state of the IAP LTR [17],[49]. In a recent study, Horie et al [50] identified transcripts from 11 loci in 129 strain embryonic stem cells that initiate in an IAP LTR and read into flanking sequence, in five cases giving rise to chimeric RNAs between an intronic IAP and the enclosing gene. In six of the 11 loci analyzed, the IAP element was not present in the B6 genome, prompting the authors to postulate that variations in IAPs may contribute to strain-specific traits [50]. We have not yet functionally examined any cases of polymorphic IAPs identified here to look for LTR-initiated fusion gene transcripts, but it is likely that numerous such cases exist.

### Concluding Remarks

Although mice and humans have similar overall numbers of old retroviral-related sequences in their genomes [2], recent levels of activity of these elements are vastly different in the two species. In humans, only about a dozen ERV loci are known to be polymorphic, and no mutations due to ERV insertions have been documented [51]. In mouse, however, ERVs/LTR retrotransposons continue to retrotranspose and are a significant source of new mutations as discussed above. Here we have used the available DNA sequence from four inbred strains to conduct an assessment of the level of insertional polymorphism of the currently active IAP and ETn/MusD ERV families. Despite mapping limitations and incomplete sequence coverage, we identified 3394 IAP and 375 ETn/MusD elements that are polymorphic among the four strains, resulting in polymorphic fractions of 61.3% and 25.6%, respectively. This is the first genome-wide determination of the extent of polymorphism of these ERV families. Given that this study was based on only a few strains, the total numbers of polymorphic elements must be substantially higher and represent a large source of genetic variation among inbred strains.

Among the polymorphic copies, 623 IAPs and 72 ETn/MusD elements reside in gene introns. In all five cases of sense-oriented ETn elements in A/J introns that we examined, evidence for gene splicing disruption was found by RT-PCR and, for two genes, further evidence of lower gene expression in A/J mice was observed through surveys of microarray data. While most polymorphic ERVs likely have little effect on host genes, we found that the prevalence within genes and the intronic orientation bias exhibited by polymorphic ERV subsets enriched for the youngest elements are distinctly different from that of older elements. This observation suggests that some of the former are deleterious but have not yet been eliminated by selection due to their short time in the genome or the controlled breeding environment of laboratory mice. Indeed, new insertions of these elements could play a significant role in genetic drift and inbreeding depression of mouse lines [52]. We propose that a comprehensive effort to document ERV and other transposable element polymorphisms among multiple inbred strains would complement SNP data and greatly contribute to our understanding of mouse genetic history and genotypic and phenotypic variation.

## Materials and Methods

### Source Data

The NCBI Trace Archive (http://0-www.ncbi.nlm.nih.gov.library.vu.edu.au/Traces/trace.cgi) included a total number of 195,993,571 traces from 38 mouse strains/classes as of May 2007. However, the majority of these traces were obtained by CHIP-related resequencing techniques, which exclude most repetitive sequences. In this study, we used only sequence traces obtained by whole genome shotgun (WGS) sequencing, which are unbiased in their content of repetitive elements. Three mouse strains (A/J, DBA/2J, 129X1/SvJ) were chosen to compare to the assembled B6 genome, [version mm8 at the UCSC Genome Browser website (http://genome.ucsc.edu)], since these were the only strains with sufficient traces sequenced by shotgun-related strategies (Figure S1).

RefSeq gene annotations were retrieved from the RefGene annotation table (version mm8, April 2007) downloaded from the UCSC Genome Browser. When an ERV insertion was found in a genomic region with multiple overlapping annotations, the one with the smallest gene size was chosen to improve specificity. We also calculated the genomic coverage of annotated RefSeq genes in the mouse genome (used as the ‘expected value’ in Figure 3A) based on the same annotation table. After merging overlapping RefSeq annotations and removing redundancies, we calculated the total coverage of genic regions in the mouse genome as 31.58%.

### Design of ERV Probes and Detection of ERVs in the Assembled B6 Genome

Three types of probes were designed based on template ERV sequences (only the type-1 probe is shown in Figure 1, step A1). For IAP, probes were based on a recently inserted polymorphic IAP 1Δ1 element (accession #EU183301) [53]. For ETn/MusD, probes were based on a mutation-causing ETnII element (accession #Y17106) [54]. MusD and ETnII elements are on average over 90% identical in the regions of the probes. To capture ETnI elements, which differ from ETnII/MusD elements in the 3′ part of the LTR and 5′ internal region [21],[22], we used a representative ETnI element (accession #AC068908). As shown in Figure S2, the type-1 probe included the full-length LTR and a small fragment of the internal ERV sequence; type-2 included only the full LTR; type-3 was only the first/last 60 bp of the 5′/3′ LTR. More information about probe design is summarized in Table S1.

We conducted a survey of both ETn/MusD and IAP insertions in the B6 genome using the 60 bp type-3 probes because they are in regions of low divergence between family members (data not shown), ensuring that all ERVs of each group will be detected. The probes were aligned to the B6 genome using the WU-BLAST 2.0 program, and any hit above our cut-off threshold was scored as an ERV insertion. To keep both sensitivity and specificity as high as possible, we designed an experiment to optimize the parameters of alignment identity and length of the aligned region and the results suggested a value of 80% for both parameters. To obtain an estimation of the sizes and numbers of ETn/MusD and IAP elements, all mapped ERV fragments (LTR termini) were merged into one individual element if they met the following criteria: 1) on the same chromosome; 2) in the same orientation; 3) within 10 kb from each other.

### Detection of ERV Insertions in Test Strains

The standalone version of the WU-BLAST v2.0 program (Gish, W. 1996–2004 http://blast.wustl.edu/) was used to make local alignments between ERV probes and mouse traces in the NCBI trace archive database (step 2 in Figure 1A). Our threshold parameters for BLAST were 80% for sequence identity and 80% for length of the aligned region. A usable ERV-containing trace consists of two parts – a non-ERV flanking sequence and the target-ERV sequence. All ERV-containing traces with a flanking portion shorter than 30 bp were discarded. Once identified, a chimeric tag was constructed by taking the whole flanking portion appended with a small tail of its target-ERV sequence (Figure 1A, step 3). We required the target-ERV tail of the tag to be 1/5 of the flanking portion in length, and a maximum of 50 bp.

The ERV-containing traces were then mapped to the assembled B6 genome. Here we used the chimeric tags derived from the previous step as the input query for BLAT [55] and mapped them to the B6 genome (version mm8) (Figure 1A, step 4). We also estimated the sequencing error rate of the mouse traces as about 5% (data not shown). We therefore defined criteria for a significant mapping as follows: 1) it should be the highest mapping score among all BLAT hits; 2) the best hit should be at least 2% higher in identity and 10% longer in mapping length compared to the second hit; 3) the alignment identity between the chimeric tag and the target locus needed to be greater than 90%; 4) the length of aligned region needed to be more than 70% of the tag length. Once a significant BLAT mapping site was identified, it was straightforward to check for presence of the ERV in the B6 genome based on alignment of the small target-ERV tail of the chimeric tag. If the BLAT mapping included more than 2/3 of the target-ERV tail, it was considered a common insertion also present in B6; if the mapping included less than 1/3 of the target-ERV tail, it was scored as absent from B6. Situations in between these two boundaries were extremely rare and were discarded.

### Determining the Polymorphism Status of ERVs Present in B6 or in the Test Strains

All sequences in the B6 genome with a length of 35 bp flanking both the 5′ and 3′ end of each detectable ERV element were aligned back to the B6 genome with BLAT and only those with a unique location were retained. Next, all these 35-bp-flanking-sequences were used as queries of the WU-BLAST program and all traces from the test strains containing such flanking sequences were collected (Figure 1, step B2). A minimum identity of 90% and a minimum mapping length of 80% were required. Because of incomplete genomic coverage of traces of test strains, many ERV flanking regions in B6 have no corresponding traces in the trace archive database and, therefore, their polymorphism status could not be determined (denoted as “?” in Tables S2 and S3). However, for ERVs in B6 with unique flanking sequence found in one or more test strain traces, presence of the ERV in test strains was determined by assessing identity between the ERV sequence in B6 and the sequence adjacent to the flanking sequence in the trace of the test strain. Here we used an implementation of the Needleman-Wunsch algorithm [56] to align the two sequences. We required a minimum identity of 90% and an alignment length of at least 35 bp to score the ERV as present in the test strain.

Using a similar strategy as above, we also assessed the polymorphism status of ERV insertions found in a test strain but not in B6. Using their locations with respect to the B6 genome, probes based on flanking genomic sequences were built and the trace archive database was searched to check if traces with the same flanking sequences were present for other test strains. All qualified traces obtained from other test strains were aligned to sequences of corresponding ERV families based on the same mapping criteria used above, and the existence of such ERV elements in other test strains was determined. Here we used exemplar ERV sequences instead of using the ERV portion in the original ERV-containing traces because, for some traces, the ERV portion was too short (less than 35 bp) to make an effective alignment.

### B6 Trace Sampling and Screening Simulations

The ERV numbers found in the three test strains are lower than the numbers detected in the assembled B6 genome. Incomplete sequence coverage and the inability to map the trace to a unique location are responsible for most of the loss of detectable ERV insertions. To estimate the fraction of ERVs that were not detected in each test strain, we applied our screening method using random samples of the unassembled B6 traces and plotted an ERV detection curve based on this simulation (Figure S3). Since the sequence quality of the B6 trace archive is generally lower than that of the three test strains, the sampling process was based only on B6 traces with less than 1% “N”s. Sample trace datasets of different sizes were constructed into simulative trace databases, and the corresponding numbers of B6 ERV insertions detected with these datasets were plotted in Figure S3. Independent random sampling was applied twice for datasets smaller than 12 million traces.

A second purpose for performing the screening simulations with B6 traces was to evaluate the accuracy of our screening method. Theoretically, all insertions found in the simulation assays in the B6 traces should be detected in the B6 reference genome. However, we did find a few cases of insertions cataloged as “polymorphic”, meaning they are from the B6 traces and were mapped to a significant locus in the B6 reference genome where no such insertion was found. One of the possible explanations for this is the fact that the assembly of the B6 genome is not perfect, especially in repetitive regions. Indeed, only 49 of the 54 non-ecotropic murine leukemia viruses (MLV) known to be present in B6 can be found in the mouse B6 assembly [57]. Nonetheless, we considered all the “polymorphic” cases in each simulation assay to be false positives and derived a conservative estimation of the accuracy of our screening method, resulting in an average false discovery rate of 0.4%±0.1%.

### Experimental Verification of Polymorphic Insertions

The presence of an insertion was tested by amplifying genomic DNA from the following strains: SWR/J, C3H/HeJ, Balb/cJ, B6, A/J, DBA/2J, 129X1/SvJ and A/WySn. All strains or DNA were from the Jackson Laboratory. Primers (see Table S4) flanking the potential insertion sites were used to amplify specific sequences from 75 ng of genomic DNA in a 25ul reaction with Phusion DNA polymerase (New England Biolabs). Cycling conditions were as per the manufacturer's instructions with annealing temperatures of between 55–65°C and extension times between 20 seconds and 4 minutes. PCR products were visualized on agarose gels. In some cases, amplification with the flanking primers did not produce a product, so one flanking primer and one LTR primer was used to confirm presence of an insertion. Therefore, in these cases, the size of the ERV insertion could not be estimated. In two cases, marked as “F” in Table 1, the PCRs were unsuccessful in one of the strains, suggesting a structural rearrangement or the presence of other polymorphisms that prevented amplification with the primers used. Some products were sequenced directly on Minelute (Qiagen) gel purified PCR fragments using the BigDye Terminator v3.1 Cycle Sequencing Kit (ABI) in an ABI PRISM® 3730XL DNA Analyzer system.

### RT-PCR

RNA from mouse tissues was extracted using RNeasy RNA isolation kit (Qiagen) according to manufacturer's recommendations. The presence of native transcripts using primers located in exons flanking the intron with the ETn insertion was confirmed with the following primer pairs: Col4a6-up-ex-s and Col4a6-down-ex-as; Dnajc10-up-ex-s and Dnajc10-down-ex-as; Mtm1-up-ex-s and Mtm1-down-ex-as; Opcml-up-ex-s and Opcml-down-ex-as; Prkca-up-ex-s and Prkca-down-ex-as. Then, RT-PCRs designed to look for chimeric transcripts between gene exons and the intronic ETn were performed. To search for transcripts utilizing the 2^nd^ and 3^rd^ splice acceptor sites in the LTR (see Figure 6), cDNA from A/J tissues specified in parentheses was amplified using a common ETn primer located downstream of the LTR, IM_3as, and the following upper exon-specific primers: Col4a6-up-ex-s (eye), Dnajc10-up-ex-s (testis), Mtm1-up-ex-s (lung), Opcml-up-ex-s (cerebral hemisphere) and Prkca-up-ex-s (eye). The same exon-specific primers and cDNA were used for the search of transcripts utilizing the first splice acceptor site, this time with the LTR-specific primer located upstream of the first PolyA site, MusD2_7130as. For *Dnajc10*, an additional PCR was performed with an upstream exon primer and a primer located at the very end of the LTR, IM_LTR_2as.

Semi-quantitative RT-PCR for the *Opcml* gene was performed with a series of A/J and B6 cerebral hemisphere cDNA dilutions, using primers in the exons upstream and downstream of the intronic ETn insertion, Opcml-ex2-s and Opcml-ex3-as. For *Gapdh*, primers Gapdh_ex6F and Gapdh_ex7R were used. *Opcml* and *Gapdh* fragments were amplified from cDNA dilutions of 1/20, 1/40 and 1/80 (Figure S7A). For each dilution, the intensity of the resulting band was quantified using ImageQuant LT (GE Healthcare) software and graphed as the intensity of *Opcml* relative to *Gapdh* (Figure S7B). The average and standard deviation among all experiments are displayed (Figure 7B). All primer sequences for RT-PCR experiments are listed in Table S4.

### Northern Blotting

RNA from A/J and B6 cerebellum was used. For each lane, 6 mg of RNA was denatured, electrophoresed in 1% agarose 3.7% formaldehyde gel in 1×MOPS buffer, transferred overnight to a Zeta-probe nylon membrane (Bio-Rad) and baked at 80°C. A probe specific for the *Opcml* exon upstream of the ETn insertion was synthesized by PCR using primers Opcml-ex2-s and Opcml-ex2-as and labeled with ^32^P using a Random Primers DNA Labeling System (Invitrogen). Membranes were prehybridized in ExpressHyb (BD Biosciences) for 4 hours at 68°C, hybridized overnight at the same temperature in fresh ExpressHyb, washed according to manufacturer's instructions and exposed to film.

### Microarray Analysis

We obtained mRNA expression microarray data of Zapala et al [41] (NCBI GEO accession GSE3594) and considered 10 tissues profiled in A/J, B6, C3H/HeJ, DBA/2J and 129S6/SvEvTac mice. We averaged the expression values for a given probeset replicated within the same strain and tissue and examined the probeset expression rank in two ways. First, we determined each strain's expression rank across genes within a given tissue, and second, the inserter strain's expression rank for a given gene was determined across tissues.

### Accession Numbers

The National Center for Biotechnology Information (NCBI) Nucleotide database (http://www.ncbi.nlm.nih.gov/sites/entrezdbNucleotide) accession number for the ETnII element used for probe design and to align in Figures 5 and S4 is Y17106. The ETnI element used for probe design is located in a BAC clone with accession number AC068908. The accession number for the IAP element used in probe design is EU183301.

## Supporting Information

Table S1 Details of ERV probes
(0.05 MB DOC)

Table S2 Polymorphic IAP cases in genes
(0.13 MB XLS)

Table S3 Polymorphic ETn/MusD cases in genes
(0.03 MB XLS)

Table S4 Primer sequences
(0.02 MB XLS)

Figure S1 Mouse trace sequence archive composition (May 2007). Numbers of sequence traces produced by whole genome shotgun methods are shown in yellow, traces produced by a re-sequencing CHIP technology shown in blue and other methods shown in green.
(0.26 MB TIF)

Figure S2 Design of probes. Type-1 probes include the full-length LTR and a small 23 bp fragment of the internal 5′ or 3′ ERV sequence. Type-2 probes consist only of the full LTR. Type-3 probes cover only the first/last 60 bp of the 5′/3′ LTR.
(0.21 MB TIF)

Figure S3 Estimation of fractional loss of detection of ERVs using sequence traces. The graph shows the fraction of ERVs present in the assembled B6 genome that were found using raw sequence traces from B6. Varying numbers of whole genome shotgun traces from B6 were mapped back to the assembled genome to detect ETn/MusD sequences as described in Materials and Methods. Arrows show the fraction of insertions found with an equivalent number of WGS traces as that available for each test strain. Standard deviations in percentages are plotted but are too small to see, the largest being 1% for the sample size of four million traces.
(0.18 MB TIF)

Figure S4 Splice sites in ETn elements detected in chimeric transcripts. An alignment of an ETnI element (chr2:110,262,865–110,268,371 of mm8 version of B6 genome) and an ETnII element is shown. The 5′ LTR and part of the 3′ LTR are shown with some interior sequence. Filled arrows indicate SA sites identified in cases of ETn mutations in the literature and also found here. Open arrows show SA sites newly identified in this study. The open triangle shows a SD site in ETnI newly identified here. PolyA site marks a polyadenylation site in some published cases. The sequence of the Dnajc10 ETnII insertion differs from that of the ETnII shown, two C-to-A mutations producing new SA sites, one suspected, marked with a “?”, another one sequenced. An ETnII element harboring these and one other mutation present in the Dnajc10 insertion is present in the B6 genome on chr13:23,177,615–23,184,720. Locations of primers used are shown with arrowed lines.
(1.29 MB TIF)

Figure S5 Comparative microarray expression data of Dnajc10 in different strains. Graphs of relative transcript levels are computer screen shots of datasets available on the GeneNetwork site (www.GeneNetwork.org). Line at the top represents the intron/exon structure of the gene with location of the ETn insertion in A/J indicated and position of the probe used for microarrays shown. A) Dataset from The Hippocampus Consortium M430v2 (Dec. 2005) RMA (Robust Analysis of Microarrays) series. Mouse strains are 129S1/Svlmj, A/J, AKR/J, Balb/cJ, C3H/HeJ, C57BL/6J, CAST/Ei, DBA/2J, KK/HIJ, LG/J, NOD/LtJ, NZO/HILtJ, PWD/PhJ, PWK/PhJ, WSB/EiJ, B6D2F1, D2B6F1. B) Dataset from the Hamilton Eye Institute mouse eye M430v2 (Nov. 2005) RMA series. Mouse strains are 129S1/Svlmj, A/J, BALB/cByJ, C3H/HeJ, C57BL/6J, CAST/EiJ, DBA/2J, KK/HIJ, LG/J, NOD/LtJ, NZO/HILtJ, PWD/PhJ, PWK/PhJ, WSB/EiJ, B6D2F1, D2B6F1. C) Dataset from the Univ. of Colorado at Denver and Health Sciences Center whole brain M430v2 (Nov06) RMA series. Mouse strains are 129P3/J, 129S1/Svlmj, A/J, AKR/J, BALB/cByJ, BALB/cJ, C3H/HeJ, C57BL/6J, C58/J, CAST/EiJ, CBA/J, DBA/2J, FVB/NJ, KK/HIJ, MOLF/EiJ, NOD/LtJ, NZW/LacJ, PWD/PhJ, SJL/J. D) Dataset from the GE-NIAAA (National Institute on Alcohol Abuse and Alcoholism) cerebellum Affymetrix M430v2 (May05) PDNN (Probe Dependent Nearest Neighbors) series. Mouse strains are 129S1/Svlmj, A/J, AKR/J, BALB/cByJ, BALB/cJ, C3H/HeJ, C57BL/6J, CAST/EiJ, DBA/2J, KK/HIJ, LG/J, NOD/LtJ. Values for A/J in each graph are marked with a star and fall significantly below the normal distribution displayed by all other strains.
(1.13 MB TIF)

Figure S6 Comparative microarray expression data of Opcml in different strains. Graphs of relative transcript levels are computer screen shots of datasets available on the GeneNetwork site (www.GeneNetwork.org). Line at the top represents the intron/exon structure of the gene with location of the ETn insertion in A/J indicated and position of the probe used for microarrays shown. A) Dataset from The Hippocampus Consortium M430v2 (Dec. 2005) RMA (Robust Analysis of Microarrays) series. B) Dataset from the Hamilton Eye Institute mouse eye M430v2 (Nov. 2005) RMA series. C) Dataset from the Univ. of Colorado at Denver and Health Sciences Center whole brain M430v2 (Nov06) RMA series. D) Dataset from the GE-NIAAA (National Institute on Alcohol Abuse and Alcoholism) cerebellum Affymetrix M430v2 (May05) PDNN (Probe Dependent Nearest Neighbors) series. Mouse strains for all datasets are listed in the legend to Figure S5. Values for A/J in each graph are marked with a star and fall significantly below the normal distribution displayed by all other strains.
(1.07 MB TIF)

Figure S7 Semi-quantitative RT-PCR of Opcml in A/J versus B6. A) Semi-quantitative RT-PCR. Opcml cDNA was amplified with primers from upstream and downstream of the ETn insertion. Opcml and Gapdh fragments were amplified from undiluted cDNA and dilutions of 1/20, 1/40 and1/80. B) Graphical representation of RT-PCR. For each dilution, the intensity of the resulting band was quantified and graphed as transcript levels of Opcml relative to Gapdh. The results of one of two representative experiments are shown.
(0.53 MB TIF)

## References

[1] Peters LL, Robledo RF, Bult CJ, Churchill GA, Paigen BJ (2007). The mouse as a model for human biology: a resource guide for complex trait analysis.. Nat Rev Genet.

[2] Waterston RH, Lindblad-Toh K, Birney E, Rogers J, Abril JF (2002). Initial sequencing and comparative analysis of the mouse genome.. Nature.

[3] Wade CM, Daly MJ (2005). Genetic variation in laboratory mice.. Nat Genet.

[4] Frazer KA, Eskin E, Kang HM, Bogue MA, Hinds DA (2007). A sequence-based variation map of 8.27 million SNPs in inbred mouse strains.. Nature.

[5] Yang H, Bell TA, Churchill GA, Pardo-Manuel d, V (2007). On the subspecific origin of the laboratory mouse.. Nat Genet.

[6] Graubert TA, Cahan P, Edwin D, Selzer RR, Richmond TA (2007). A high-resolution map of segmental DNA copy number variation in the mouse genome.. PLos Genet.

[7] Li J, Jiang T, Mao JH, Balmain A, Peterson L (2004). Genomic segmental polymorphisms in inbred mouse strains.. Nat Genet.

[8] Maksakova I, Romanish M, Gagnier L, Dunn CA, Mager DL (2006). Retroviral elements and their hosts: Insertional mutagenesis in the mouse germ line.. PLoS Genetics.

[9] Boeke JD, Stoye JP, Coffin JM, Hughes SH, Varmus HE (1997). Retrotransposons, endogenous retroviruses, and the evolution of retrolements.. Retroviruses.

[10] Stoye JP, Coffin JM (1988). Polymorphism of murine endogenous proviruses revealed by using virus class-specific oligonucleotide probes.. J Virol.

[11] Frankel WN, Stoye JP, Taylor BA, Coffin JM (1990). A linkage map of endogenous murine leukemia proviruses.. Genetics.

[12] Kung HJ, Boerkoel C, Carter TH (1991). Retroviral mutagenesis of cellular oncogenes: a review with insights into the mechanisms of insertional activation.. Curr Top Microbiol Immunol.

[13] Rosenberg N, Jolicoeur P, Coffin JM, Hughes SH, Varmus HE (1997). Retroviral pathogenesis.. Retroviruses.

[14] Dudley JP (2003). Tag, you're hit: retroviral insertions identify genes involved in cancer.. Trends Mol Med.

[15] Theodorou V, Kimm MA, Boer M, Wessels L, Theelen W (2007). MMTV insertional mutagenesis identifies genes, gene families and pathways involved in mammary cancer.. Nat Genet.

[16] Kuff EL, Lueders KK (1988). The intracisternal A-particle gene family: Structural and functional aspects.. Adv Cancer Res.

[17] Druker R, Whitelaw E (2004). Retrotransposon-derived elements in the mammalian genome: a potential source of disease.. J Inherit Metab Dis.

[18] Wang XY, Steelman LS, McCubrey JA (1997). Abnormal activation of cytokine gene expression by intracisternal type A particle transposition: effects of mutations that result in autocrine growth stimulation and malignant transformation.. Cytokines Cell Mol Ther.

[19] Brulet P, Condamine H, Jacob F (1985). Spatial distribution of transcripts of the long repeated ETn sequence during early mouse embryogenesis.. Proc Natl Acad Sci USA.

[20] Loebel DA, Tsoi B, Wong N, O'Rourke MP, Tam PP (2004). Restricted expression of ETn-related sequences during post-implantation mouse development.. Gene Expr Patterns.

[21] Shell BE, Collins JT, Elenich LA, Szurek PF, Dunnick WA (1990). Two subfamilies of murine retrotransposon ETn sequences.. Gene.

[22] Baust C, Gagnier L, Baillie GJ, Harris MJ, Juriloff DM (2003). Structure and expression of mobile ETnII retroelements and their coding-competent MusD relatives in the mouse.. J Virol.

[23] Mager DL, Freeman JD (2000). Novel mouse type D endogenous proviruses and ETn elements share long terminal repeat and internal sequences.. J Virol.

[24] Ribet D, Dewannieux M, Heidmann T (2004). An active murine transposon family pair: retrotransposition of “master” MusD copies and ETn *trans*-mobilization.. Genome Res.

[25] Kaushik N, Stoye JP (1994). Intracisternal A-type particle elements as genetic markers: detection by repeat element viral element amplified locus-PCR.. Mamm Genome.

[26] Lueders KK, Frankel WN (1994). Mapping of mouse intracisternal A-particle proviral markers in an interspecific backcross.. Mamm Genome.

[27] Lueders KK, Frankel WN, Mietz JA, Kuff EL (1993). Genomic mapping of intracisternal A-particle proviral elements.. Mamm Genome.

[28] Baust C, Baillie GJ, Mager DL (2002). Insertional polymorphisms of ETn retrotransposons include a disruption of the *wiz* gene in C57BL/6 mice.. Mamm Genome.

[29] Mager DL, Medstrand P (2003). Retroviral repeat sequences.. Nature Encyclopedia of the Human Genome.

[30] Smit AF (1999). Interspersed repeats and other mementos of transposable elements in mammalian genomes.. Curr Opin Genet Dev.

[31] Medstrand P, van de Lagemaat LN, Mager DL (2002). Retroelement distributions in the human genome: variations associated with age and proximity to genes.. Genome Res.

[32] van de Lagemaat LN, Medstrand P, Mager DL (2006). Multiple effects govern endogenous retrovirus survival patterns in human gene introns.. Genome Biol.

[33] Cutter AD, Good JM, Pappas CT, Saunders MA, Starrett DM (2005). Transposable element orientation bias in the Drosophila melanogaster genome.. J Mol Evol.

[34] Barr SD, Leipzig J, Shinn P, Ecker JR, Bushman FD (2005). Integration targeting by avian sarcoma-leukosis virus and human immunodeficiency virus in the chicken genome.. J Virol.

[35] Mitchell RS, Beitzel BF, Schroder AR, Shinn P, Chen H (2004). Retroviral DNA integration: ASLV, HIV, and MLV show distinct target site preferences.. PLoS Biol.

[36] Dewannieux M, Dupressoir A, Harper F, Pierron G, Heidmann T (2004). Identification of autonomous IAP LTR retrotransposons mobile in mammalian cells.. Nat Genet.

[37] Beck JA, Lloyd S, Hafezparast M, Lennon-Pierce M, Eppig JT (2000). Genealogies of mouse inbred strains.. Nat Genet.

[38] Ho M, Post CM, Donahue LR, Lidov HG, Bronson RT (2004). Disruption of muscle membrane and phenotype divergence in two novel mouse models of dysferlin deficiency.. Hum Mol Genet.

[39] Maksakova IA, Mager DL (2005). Transcriptional regulation of early transposon elements, an active family of mouse long terminal repeat retrotransposons.. J Virol.

[40] Barrett T, Troup DB, Wilhite SE, Ledoux P, Rudnev D (2007). NCBI GEO: mining tens of millions of expression profiles–database and tools update.. Nucleic Acids Res.

[41] Zapala MA, Hovatta I, Ellison JA, Wodicka L, Del Rio JA (2005). Adult mouse brain gene expression patterns bear an embryologic imprint.. Proc Natl Acad Sci USA.

[42] Cunnea PM, Miranda-Vizuete A, Bertoli G, Simmen T, Damdimopoulos AE (2003). ERdj5, an endoplasmic reticulum (ER)-resident protein containing DnaJ and thioredoxin domains, is expressed in secretory cells or following ER stress.. J Biol Chem.

[43] Corazzari M, Lovat PE, Armstrong JL, Fimia GM, Hill DS (2007). Targeting homeostatic mechanisms of endoplasmic reticulum stress to increase susceptibility of cancer cells to fenretinide-induced apoptosis: the role of stress proteins ERdj5 and ERp57.. Br J Cancer.

[44] Schofield PR, McFarland KC, Hayflick JS, Wilcox JN, Cho TM (1989). Molecular characterization of a new immunoglobulin superfamily protein with potential roles in opioid binding and cell contact.. EMBO J.

[45] Yamada M, Hashimoto T, Hayashi N, Higuchi M, Murakami A (2007). Synaptic adhesion molecule OBCAM; synaptogenesis and dynamic internalization.. Brain Res.

[46] Sellar GC, Watt KP, Rabiasz GJ, Stronach EA, Li L (2003). *OPCML* at 11q25 is epigenetically inactivated and has tumor-suppressor function in epithelial ovarian cancer.. Nat Genet.

[47] Reed JE, Dunn JR, du Plessis DG, Shaw EJ, Reeves P (2007). Expression of cellular adhesion molecule *‘OPCML’* is down-regulated in gliomas and other brain tumours.. Neuropathol Appl Neurobiol.

[48] Struyk AF, Canoll PD, Wolfgang MJ, Rosen CL, d'Eustachio P (1995). Cloning of neurotrimin defines a new subfamily of differentially expressed neural cell adhesion molecules.. J Neurosci.

[49] Blewitt ME, Vickaryous NK, Paldi A, Koseki H, Whitelaw E (2006). Dynamic reprogramming of DNA methylation at an epigenetically sensitive allele in mice.. PLoS Genet.

[50] Horie K, Saito ES, Keng VW, Ikeda R, Ishihara H (2007). Retrotransposons influence the mouse transcriptome: Implication for the divergence of genetic traits.. Genetics.

[51] Moyes D, Griffiths DJ, Venables PJ (2007). Insertional polymorphisms: a new lease of life for endogenous retroviruses in human disease.. Trends Genet.

[52] Taft RA, Davisson M, Wiles MV (2006). Know thy mouse.. Trends Genet.

[53] Juriloff DM, Harris MJ, Dewell SL, Brown CJ, Mager DL (2005). Investigations of the genomic region that contains the *clf1* mutation, a causal gene in multifactorial cleft lip and palate in mice.. Birth Defects Res A Clin Mol Teratol.

[54] Hofmann M, Harris M, Juriloff D, Boehm T (1998). Spontaneous mutations in SELH/Bc mice due to insertions of early transposons: molecular characterization of null alleles at the nude and albino loci.. Genomics.

[55] Kent WJ (2002). BLAT–the BLAST-like alignment tool.. Genome Res.

[56] Needleman SB, Wunsch CD (1970). A general method applicable to the search for similarities in the amino acid sequence of two proteins.. J Mol Biol.

[57] Jern P, Stoye JP, Coffin J (2007). Role of APOBEC3 in genetic diversity among endogenous murine leukemia viruses.. PLoS Genetics.

