# Supplementary material for: Genome-Wide Assessments Reveal Extremely High Levels of Polymorphism of Two Active Families of Mouse Endogenous Retroviral Elements
Source: PLoS Genet. 2008 Feb 29;4(2):e1000007. doi: 10.1371/journal.pgen.1000007 (PMC2265474; doi:10.1371/journal.pgen.1000007)
Supplement: Table S1 — Details of ERV probes (0.05 MB DOC) [file pgen.1000007.s001.doc]

Table S1: Details of ERV Probes

| Target ERV | Probe Type | Probe Name | Probe Structure | Length | Template ERV |
| --- | --- | --- | --- | --- | --- |
| ETn/MusD | Type-1 | probe1_5p | 5’ LTR + ERV internal | 340 bp | Y17106^a^ (ETnII^b^) |
|  |  | probe1_3p | ERV internal + 3’ LTR | 340 bp | Y17106 (ETnII) |
|  | Type-2 | probe2 | full LTR | 317 bp | Y17106 (ETnII) |
|  | Type-3 | Probe3_U3 | 5’-end of the LTR | 60 bp | Y17106 (ETnII) |
|  |  | Probe3_U5A | 3’-end of the LTR | 60 bp | Y17106 (ETnII) |
|  |  | Probe3_U5B | 3’-end of the LTR | 60 bp | AC068908^a^ (ETnI^b^) |
| IAP | Type-1 | probe1_5p | 5’ LTR + ERV internal | 376 bp | EU183301^a^ (IΔ1^c^) |
|  |  | probe1_3p | ERV internal + 3’ LTR | 376 bp | EU183301 (IΔ1) |
|  | Type-2 | probe2 | full LTR | 317 bp | EU183301 (IΔ1) |
|  | Type-3 | Probe3_U3 | 5’-end of the LTR | 60 bp | EU183301 (IΔ1) |
|  |  | Probe3_U5 | 3’-end of the LTR | 60 bp | EU183301 (IΔ1) |

^a^Accession numbers of ERVs used for probe design.

^b^ETnI and ETnII are different ETn/MusD subfamilies

^c^IΔ1 is an IAP subfamily that is currently the most active in mice
