# Supplementary figures and images for: Genome-Wide Assessments Reveal Extremely High Levels of Polymorphism of Two Active Families of Mouse Endogenous Retroviral Elements
Source: PLoS Genet. 2008 Feb 29;4(2):e1000007. doi: 10.1371/journal.pgen.1000007 (PMC2265474; doi:10.1371/journal.pgen.1000007)

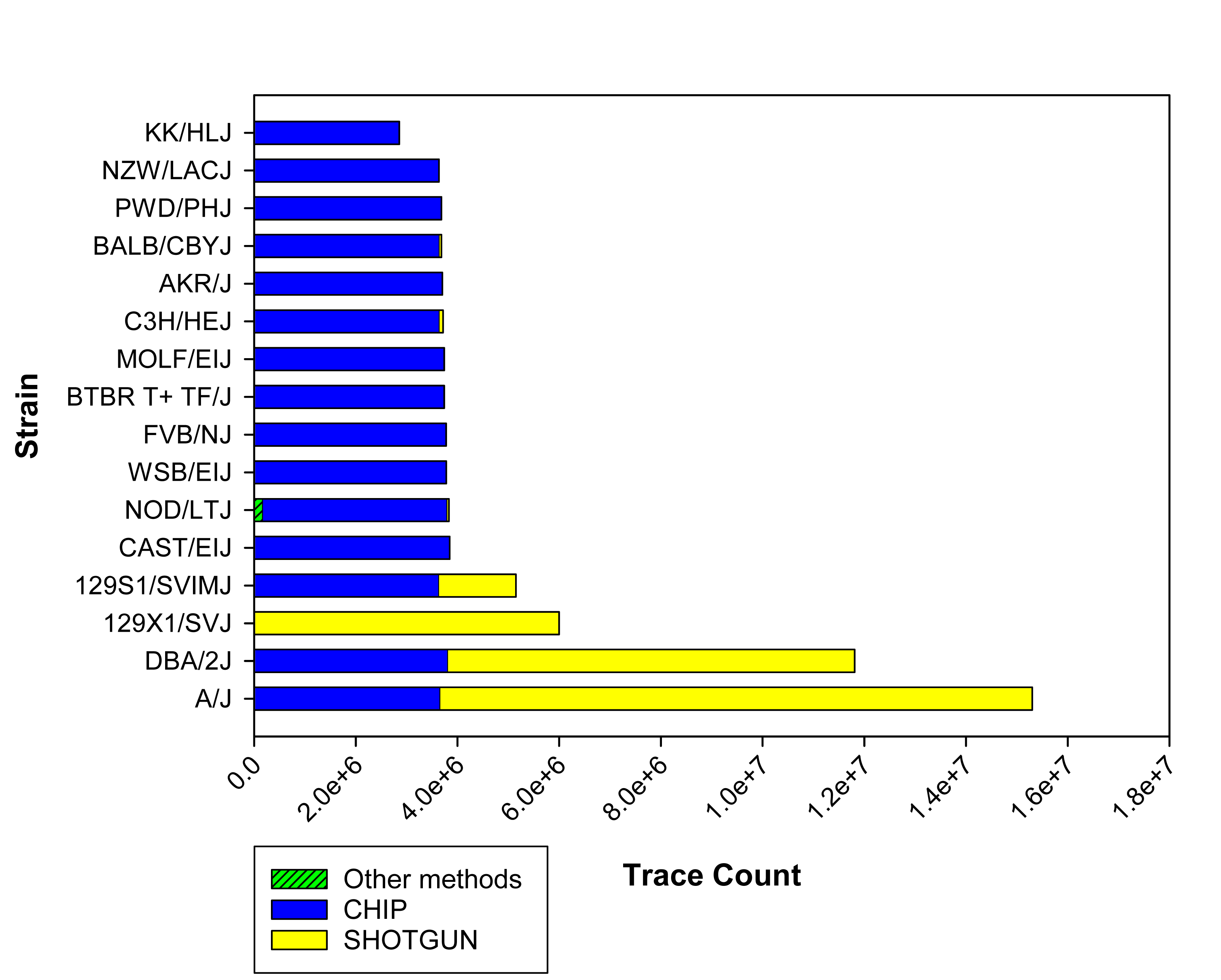

Supplement: Figure S1 — Mouse trace sequence archive composition (May 2007). Numbers of sequence traces produced by whole genome shotgun methods are shown in yellow, traces produced by a re-sequencing CHIP technology shown in blue and other methods shown in green. (0.26 MB TIF) [file pgen.1000007.s005.tif]

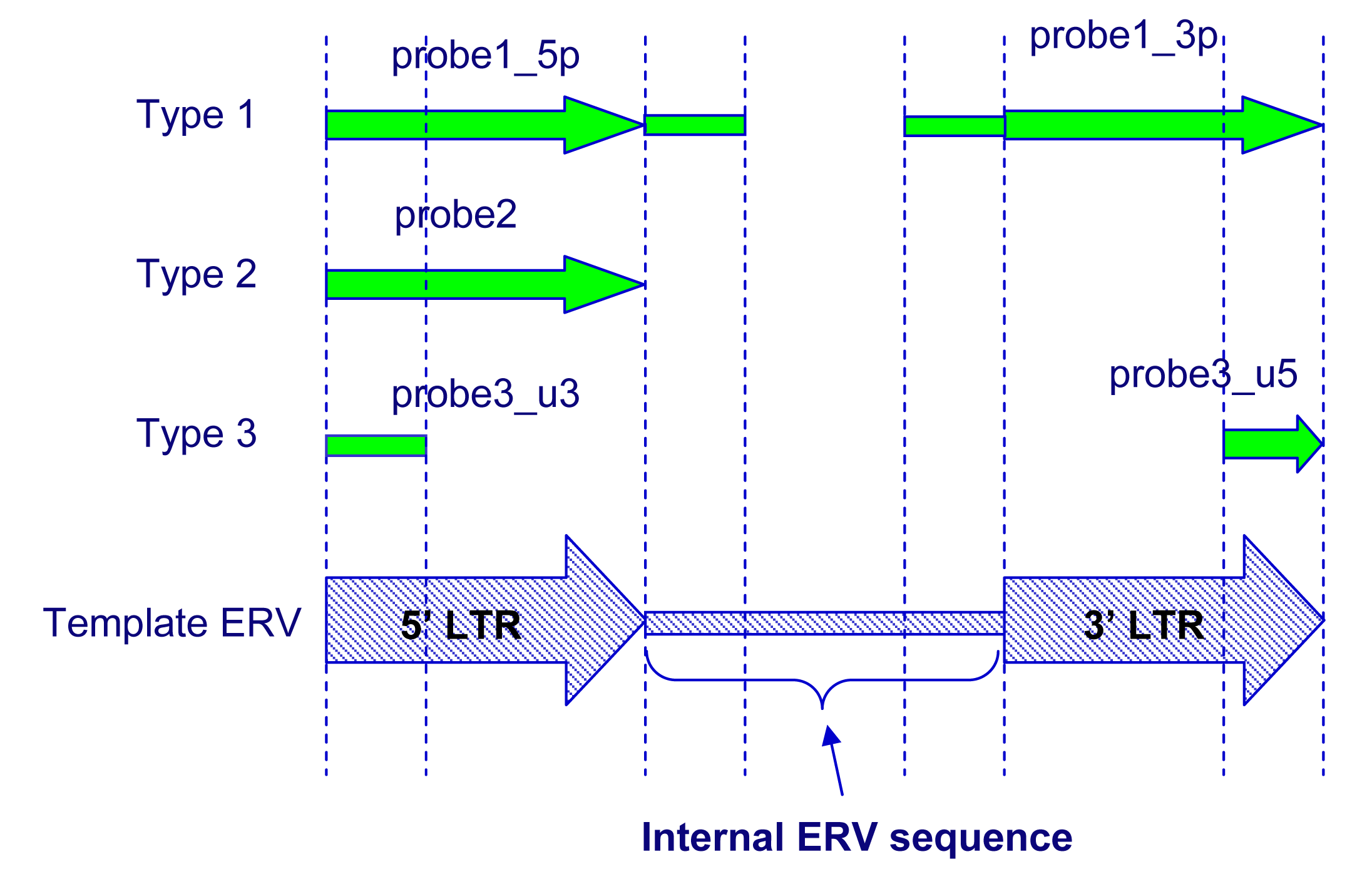

Supplement: Figure S2 — Design of probes. Type-1 probes include the full-length LTR and a small 23 bp fragment of the internal 5′ or 3′ ERV sequence. Type-2 probes consist only of the full LTR. Type-3 probes cover only the first/last 60 bp of the 5′/3′ LTR. (0.21 MB TIF) [file pgen.1000007.s006.tif]

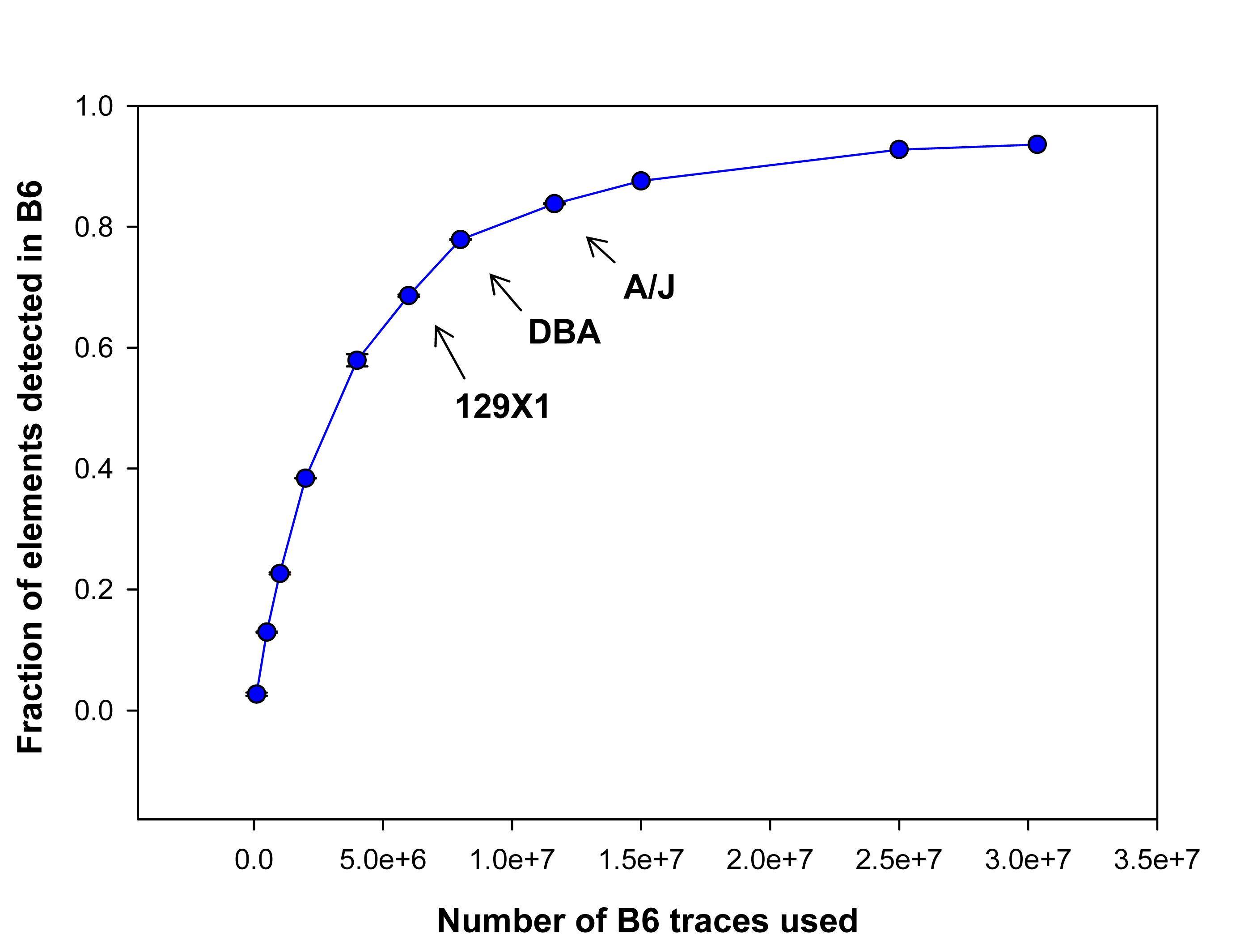

Supplement: Figure S3 — Estimation of fractional loss of detection of ERVs using sequence traces. The graph shows the fraction of ERVs present in the assembled B6 genome that were found using raw sequence traces from B6. Varying numbers of whole genome shotgun traces from B6 were mapped back to the assembled genome to detect ETn/MusD sequences as described in Materials and Methods. Arrows show the fraction of insertions found with an equivalent number of WGS traces as that available for each test strain. Standard deviations in percentages are plotted but are too small to see, the largest being 1% for the sample size of four million traces. (0.18 MB TIF) [file pgen.1000007.s007.tif]

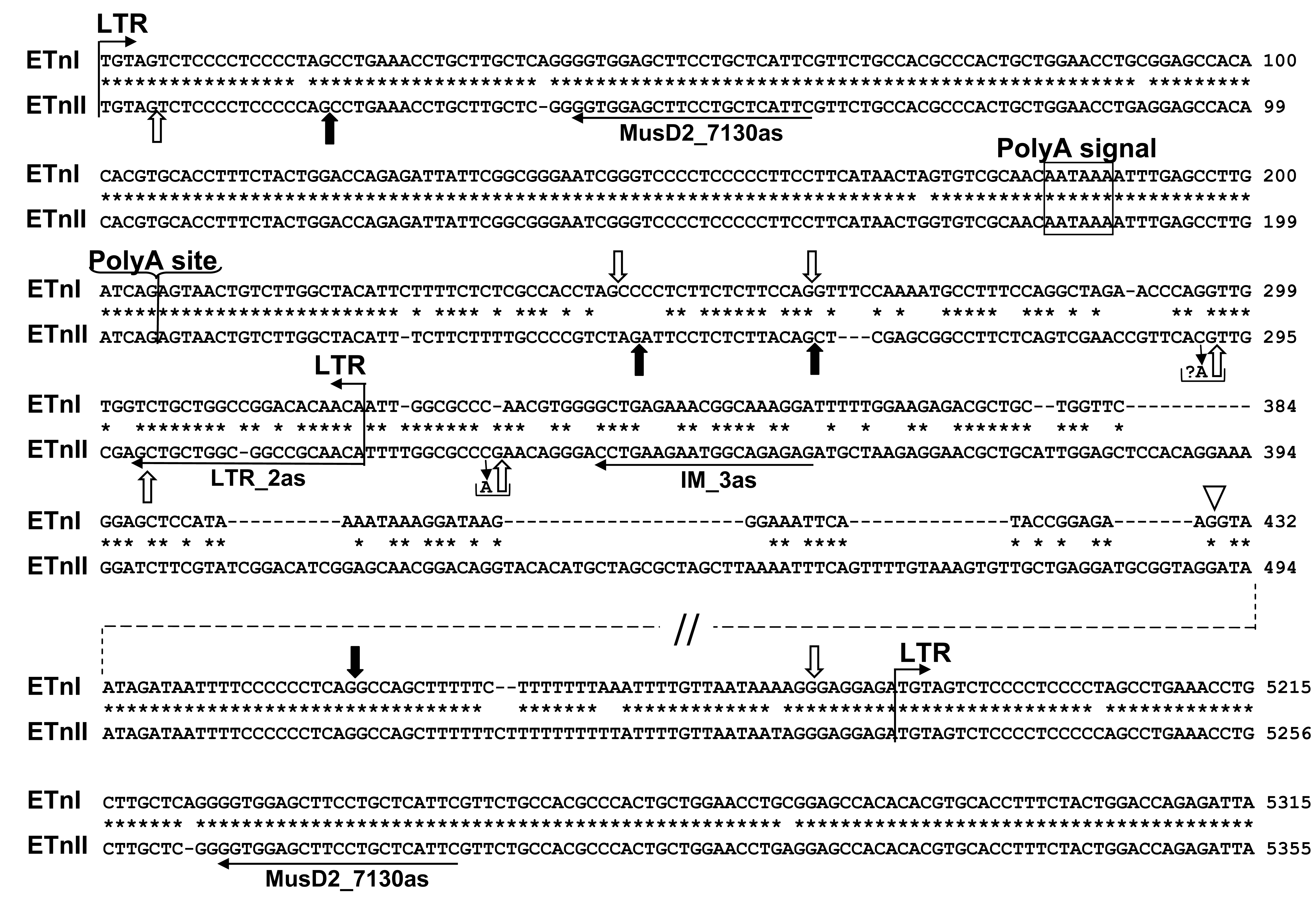

Supplement: Figure S4 — Splice sites in ETn elements detected in chimeric transcripts. An alignment of an ETnI element (chr2:110,262,865–110,268,371 of mm8 version of B6 genome) and an ETnII element is shown. The 5′ LTR and part of the 3′ LTR are shown with some interior sequence. Filled arrows indicate SA sites identified in cases of ETn mutations in the literature and also found here. Open arrows show SA sites newly identified in this study. The open triangle shows a SD site in ETnI newly identified here. PolyA site marks a polyadenylation site in some published cases. The sequence of the Dnajc10 ETnII insertion differs from that of the ETnII shown, two C-to-A mutations producing new SA sites, one suspected, marked with a “?”, another one sequenced. An ETnII element harboring these and one other mutation present in the Dnajc10 insertion is present in the B6 genome on chr13:23,177,615–23,184,720. Locations of primers used are shown with arrowed lines. (1.29 MB TIF) [file pgen.1000007.s008.tif]

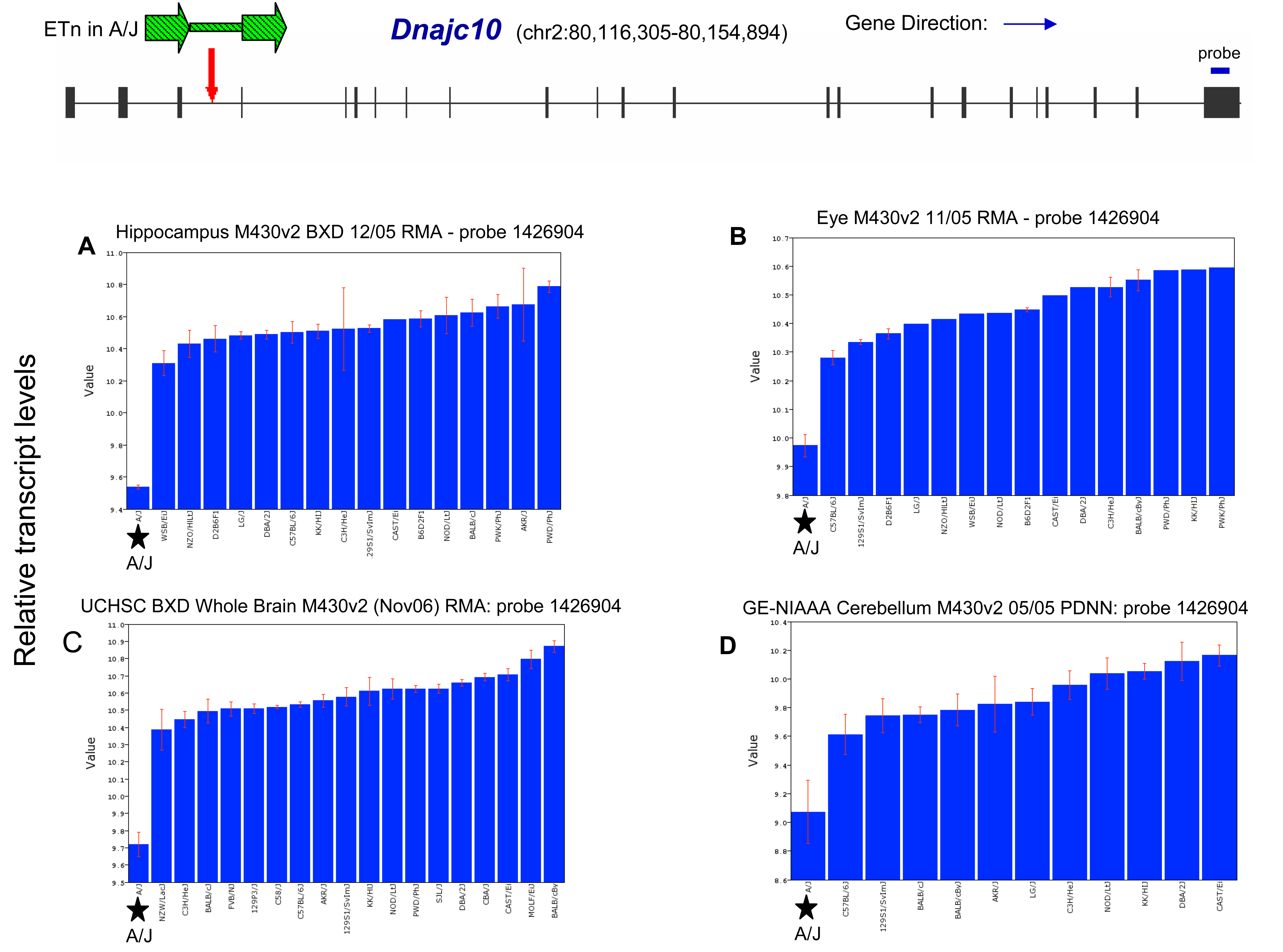

Supplement: Figure S5 — Comparative microarray expression data of Dnajc10 in different strains. Graphs of relative transcript levels are computer screen shots of datasets available on the GeneNetwork site (www.GeneNetwork.org). Line at the top represents the intron/exon structure of the gene with location of the ETn insertion in A/J indicated and position of the probe used for microarrays shown. A) Dataset from The Hippocampus Consortium M430v2 (Dec. 2005) RMA (Robust Analysis of Microarrays) series. Mouse strains are 129S1/Svlmj, A/J, AKR/J, Balb/cJ, C3H/HeJ, C57BL/6J, CAST/Ei, DBA/2J, KK/HIJ, LG/J, NOD/LtJ, NZO/HILtJ, PWD/PhJ, PWK/PhJ, WSB/EiJ, B6D2F1, D2B6F1. B) Dataset from the Hamilton Eye Institute mouse eye M430v2 (Nov. 2005) RMA series. Mouse strains are 129S1/Svlmj, A/J, BALB/cByJ, C3H/HeJ, C57BL/6J, CAST/EiJ, DBA/2J, KK/HIJ, LG/J, NOD/LtJ, NZO/HILtJ, PWD/PhJ, PWK/PhJ, WSB/EiJ, B6D2F1, D2B6F1. C) Dataset from the Univ. of Colorado at Denver and Health Sciences Center whole brain M430v2 (Nov06) RMA series. Mouse strains are 129P3/J, 129S1/Svlmj, A/J, AKR/J, BALB/cByJ, BALB/cJ, C3H/HeJ, C57BL/6J, C58/J, CAST/EiJ, CBA/J, DBA/2J, FVB/NJ, KK/HIJ, MOLF/EiJ, NOD/LtJ, NZW/LacJ, PWD/PhJ, SJL/J. D) Dataset from the GE-NIAAA (National Institute on Alcohol Abuse and Alcoholism) cerebellum Affymetrix M430v2 (May05) PDNN (Probe Dependent Nearest Neighbors) series. Mouse strains are 129S1/Svlmj, A/J, AKR/J, BALB/cByJ, BALB/cJ, C3H/HeJ, C57BL/6J, CAST/EiJ, DBA/2J, KK/HIJ, LG/J, NOD/LtJ. Values for A/J in each graph are marked with a star and fall significantly below the normal distribution displayed by all other strains. (1.13 MB TIF) [file pgen.1000007.s009.tif]

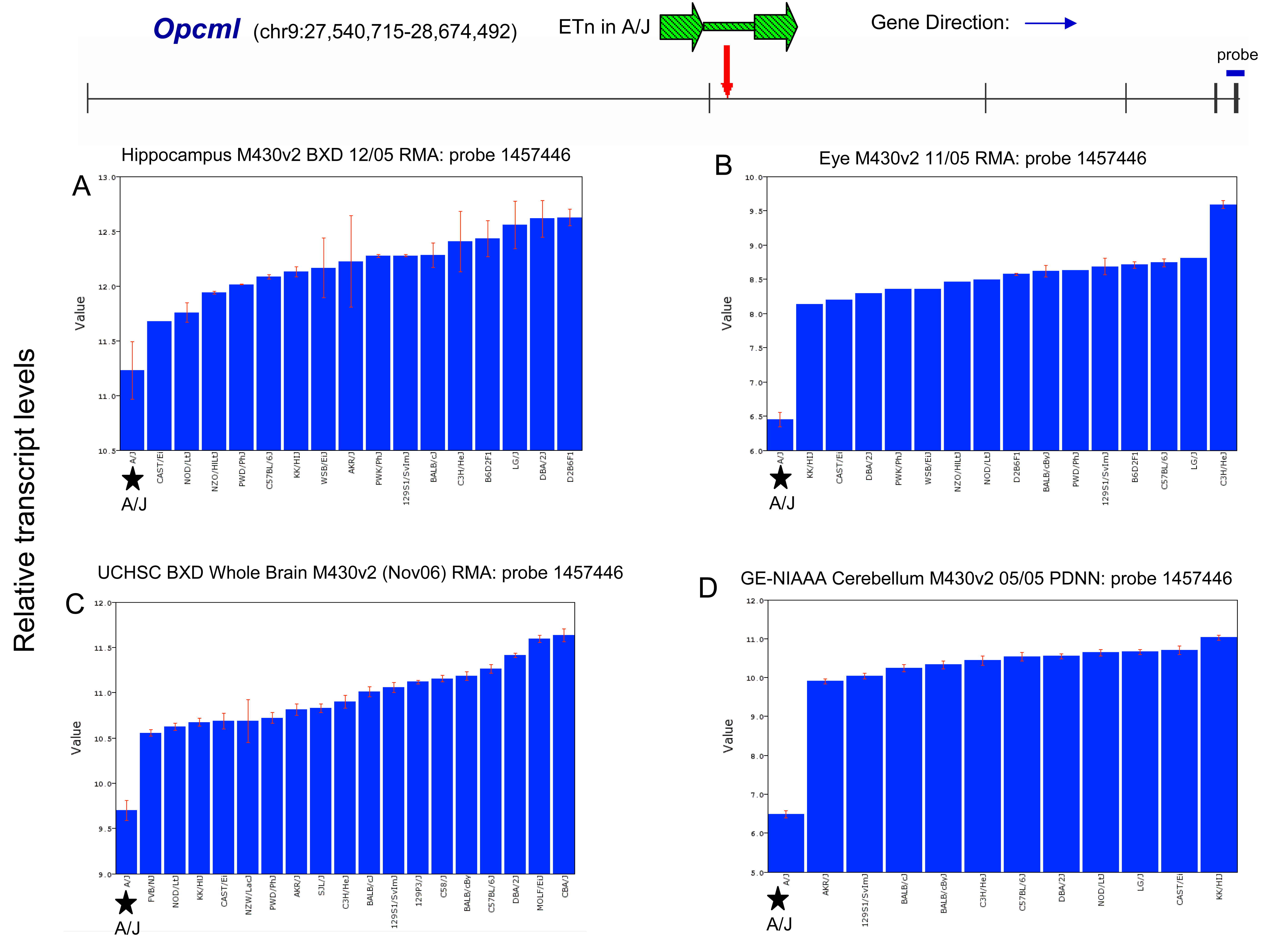

Supplement: Figure S6 — Comparative microarray expression data of Opcml in different strains. Graphs of relative transcript levels are computer screen shots of datasets available on the GeneNetwork site (www.GeneNetwork.org). Line at the top represents the intron/exon structure of the gene with location of the ETn insertion in A/J indicated and position of the probe used for microarrays shown. A) Dataset from The Hippocampus Consortium M430v2 (Dec. 2005) RMA (Robust Analysis of Microarrays) series. B) Dataset from the Hamilton Eye Institute mouse eye M430v2 (Nov. 2005) RMA series. C) Dataset from the Univ. of Colorado at Denver and Health Sciences Center whole brain M430v2 (Nov06) RMA series. D) Dataset from the GE-NIAAA (National Institute on Alcohol Abuse and Alcoholism) cerebellum Affymetrix M430v2 (May05) PDNN (Probe Dependent Nearest Neighbors) series. Mouse strains for all datasets are listed in the legend to Figure S5. Values for A/J in each graph are marked with a star and fall significantly below the normal distribution displayed by all other strains. (1.07 MB TIF) [file pgen.1000007.s010.tif]

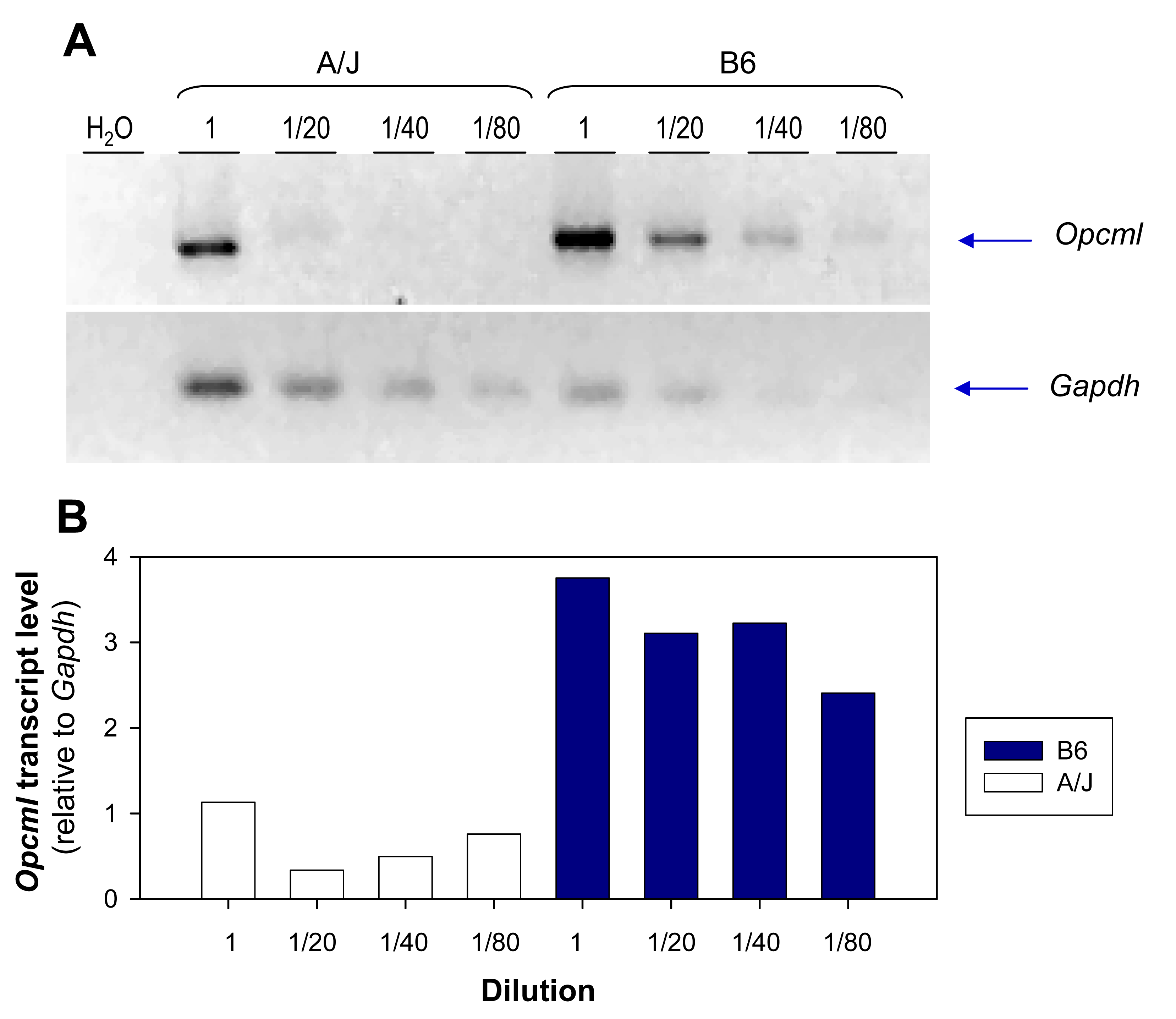

Supplement: Figure S7 — Semi-quantitative RT-PCR of Opcml in A/J versus B6. A) Semi-quantitative RT-PCR. Opcml cDNA was amplified with primers from upstream and downstream of the ETn insertion. Opcml and Gapdh fragments were amplified from undiluted cDNA and dilutions of 1/20, 1/40 and1/80. B) Graphical representation of RT-PCR. For each dilution, the intensity of the resulting band was quantified and graphed as transcript levels of Opcml relative to Gapdh. The results of one of two representative experiments are shown. (0.53 MB TIF) [file pgen.1000007.s011.tif]
